# Targeting Neutrophil/Eosinophil Extracellular Traps by Aptamer‐Functionalized Nanosheets to Overcome Recalcitrant Inflammatory Disorders

**DOI:** 10.1002/advs.202504210

**Published:** 2025-07-14

**Authors:** Yongqiang Xiao, Xinyue Wang, Ming Liu, Xiao Fu, Nanfeng Zhang, Wenqing Yang, Junyi Ge, Yangyang Li, Duan Ma, Jing Ma, Weiping Wen, Dongdong Ren, Tianyu Zhang, Zhaoxu Tu

**Affiliations:** ^1^ ENT Institute Department of Facial Plastic and Reconstructive Surgery Eye & ENT Hospital Fudan University Shanghai 200031 China; ^2^ Department of Otolaryngology The Sixth Affiliated Hospital Sun Yat‐sen University Guangzhou Guangdong 510655 China; ^3^ Department of Otology and Skull Base Surgery EYE and ENT Hospital of Fudan University Shanghai 200031 China; ^4^ Key Laboratory of Metabolism and Molecular Medicine Ministry of Education Department of Biochemistry and Molecular Biology School of Basic Medical Sciences Fudan University Shanghai 200032 China; ^5^ Department of Endocrinology Shuguang Hospital Affiliated to Shanghai University of Traditional Chinese Medicine Shanghai 200021 China; ^6^ Department of ENT Second Affiliated Hospital of Anhui Medical University Hefei Anhui 230031 China

**Keywords:** aptamers, copper indium thiophosphate, dysregulated inflammation, neutrophil/eosinophil extracellular traps, tannic acid

## Abstract

Excessive generation of neutrophil extracellular traps (NETs) and eosinophil extracellular traps (EETs) can drive various inflammatory disorders by stimulating intracellular nucleic acid receptors. Although extracellular traps (ETs) are promising therapeutic targets for recalcitrant chronic inflammation, for example, otitis media with effusion (OME), practical and specific targeting of NETs/EETs in pathological tissues remains challenging. In this study, an ultrathin, 2D sheet‐like nanoscavenger C‐TA_H_ is developed by modifying copper indium thiophosphate (CIPS) nanosheets with tannic acid (TA) and histone aptamers. The findings reveal that C‐TA_H_ effectively binds the dsDNA of NETs/EETs, inhibits bacterial growth, and reduces reactive oxygen species (ROS) levels, leading to a depressed local inflammation in ovalbumin (OVA)‐induced OME rats. In addition, the therapeutic results also include reductions in inflammatory cytokines release, suppression of ETs‐activated danger signaling pathways, including toll‐like receptor 9 (TLR9) and nuclear factor‐kappa B (NF‐κB), as well as decreased mucous exudation and improved hearing functions. Comprehensively, transcriptomic analysis with RNA‐sequencing confirms that C‐TA_H_ treatment significantly reverses the pathological gene expression changes after OVA sensitization. This work introduces an aptamer modification strategy for the target and capture of NETs/EETs, providing a potential therapeutic approach for modulating inflammatory signaling in OME as well as other recalcitrant inflammatory disorders.

## Graphical abstract

In this study, C‐TA_H_, as multifunctional nanosheets, is developed with tannic acid and histone aptamer coverage. C‐TA_H_ displays mild cytotoxicity, robust dsDNA and NETs/EETs binding efficiency, and potent antioxidant and antibacterial ability in vitro. C‐TA_H_ treatment ameliorates dysregulated inflammation and restores hearing function in animal models, promoting inspiration for the treatment of otitis media and other recalcitrant inflammatory disorders.

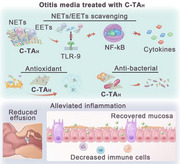

## 1 Introduction

Neutrophil extracellular traps (NETs) and eosinophil extracellular traps (EETs) are large, extracellular, web‐like chromatin structures released by stimulated neutrophils and eosinophils.^[^ ^1^ ^]^ Despite controlling pathogen infections,^[^ ^2^ ^]^ excessive NETs/EETs also contribute to dysregulated inflammatory disorders.^[^ ^3^ ^]^ Recent studies have highlighted the presence of NETs/EETs in various inflammation‐related diseases, which are implicated in exacerbating inflammatory responses.^[^ ^1^, ^4^ ^]^ In this work, otitis media with effusion (OME), a condition affecting 80–90% of preschool children with Eustachian tube dysfunction,^[^ ^5^ ^]^ was investigated. Persistent OME can lead to bacterial infections and chronic otitis media, with both surgical and non‐surgical treatments often causing complications or being ineffective.^[^ ^6^ ^]^ Without proper treatment, more serious otogenic complications, such as mastoiditis, labyrinthitis, venous sinus thrombosis, or intracranial complications, may also occur, potentially leading to fatal outcomes.^[^ ^7^ ^]^ Recent reports have also emphasized the pivotal roles of NETs/EETs in the onset and progression of otitis media, suggesting them as potential therapeutic targets.^[^ ^8^ ^]^

It is extensively recognized that the double‐stranded DNA (dsDNA) embedded in NETs/EETs can be recognized by pattern recognition receptors (PRRs) in immune cells.^[^ ^9^ ^]^ This recognition initiates an intracellular immune signaling cascade, leading to the release of pro‐inflammatory cytokines.^[^ ^10^ ^]^ Additionally, ETs hold cytotoxic effects on healthy cells and tissues, promoting local inflammation and tissue damage.^[^ ^10^ ^]^ The accumulation of NETs/EETs in blood and tissues can elicit both systemic and local inflammatory responses, which are associated with a range of inflammatory and infectious diseases.^[^ ^2^, ^11^ ^]^ Of particular concern, DNA moleculesare a key structural component of extracellular polymeric substances in bacterial biofilms, making ETs an attractive target for controlling biofilm‐related infections.^[^ ^12^ ^]^ Moreover, EETs are closely linked to allergen‐triggered type 2 immune cascade and play critical roles in many allergic inflammatory conditions.^[^ ^13^ ^]^ Given this, therapeutic strategies targeting ETs—specifically steps involved in neutrophil and eosinophil activation, migration, and stimulation—hold promise for the treatment of numerous inflammation‐related diseases. Although the removal of NETs/EETs is promising in treating ETs‐associated inflammatory disorders, there are currently no targeted therapeutic strategies in clinical settings.^[^ ^14^ ^]^ Although DNase I‐mediated degradation of NETs/EETs has been shown to alleviate severe airway inflammatory diseases, concerns regarding its stability, cost, and potential side effects still remain.^[^ ^15^ ^]^ Recently, biomaterials‐based nanomedicines have been exploited in the prevention and clinical treatment of many diseases, for instance, mRNA vaccine for COVID‐19 prevention.^[^ ^16^ ^]^ Therefore, it is extremely meaningful to invent novel NET/EET scavenging strategies with biomaterials for inflammatory diseases relevant to ETs. Previous studies displayed cationic nanomaterials as dsDNA scavengers could be used to treat many inflammatory disorders. Nevertheless, most of them are inadequate for ET elimination, attributed to the relatively large volume and intricate composition of ETs.

In our previous studies, functionalized 2D nanosheets, characterized by their sheet‐like nanostructure, high surface‐to‐volume ratio, and flexible backbone, have demonstrated therapeutic efficacy in restoring dysregulated inflammation across multiple disease models, including airway disorders,^[^ ^18^ ^]^ sepsis,^[^ ^19^ ^]^ and kidney injury.^[^ ^20^ ^]^ Importantly, these nanosheets exhibit superior therapeutic performance compared to their polymeric and nanosphere counterparts, owing to their enhanced multivalent interactions with danger signal molecules.^[^ ^20^, ^21^ ^]^ Building upon these findings, we propose that engineered 2D nanoplatforms hold significant promise for addressing challenging ET‐associated inflammatory diseases, particularly OME. Recent advances have identified copper indium thiophosphate (CuInP2S6, CIPS) nanosheets as an emerging class of 2D nanomaterials, distinguished by their broad‐spectrum antimicrobial activity, excellent storage stability, and exceptional biocompatibility.^[^ ^22^ ^]^

Tannic acid (TA), a polyphenolic compound, can form stable complexes with DNA molecules, thereby inhibiting the dsDNA‐elicited inflammatory cascade.^[^ ^17^ ^]^ In addition to dsDNA scavenging, TA‐based nanoformulations neutralize reactive oxygen species (ROS), boost antioxidant enzyme activity, reduce inflammation, and shield against oxidative stress‐related diseases.^[^ ^18^ ^]^ Nevertheless, the specific binding ability of nanoplatforms to NETs/EETs structure must be further enhanced to prepare more sophisticated anti‐inflammation nanomedicine. Recently, the aptamers, as short, single‐stranded nucleic acids, have been engineered to particularly bind to targeted proteins with high affinity.^[^ ^19^ ^]^ As some specific proteins (histone, etc.) were involved in the NET/EET structures,^[^ ^20^ ^]^ it was hypothesized that CIPS nanosheets modified with TA and particular aptamers were promising to serve as ET scavengers.

This study first substantiated a positive correlation between NET/EET levels and inflammation severity in the middle ear exudate (MEE) of OME patients. To construct effective nanoplatforms for ET scavenging, CIPS nanosheets, an ultrathin 2D backbone, were selected because of their excellent biomedical properties.^[^ ^21^ ^]^ TA and histone aptamers were engineered to fabricate TA‐covered CIPS (C‐TA) and histone aptamer‐modified C‐TA (C‐TA_H_), and their biocompatibility and binding efficacy with dsDNA and NETs/EETs were carefully evaluated. Afterward, we systematically investigated the anti‐inflammation, ROS reduction, and anti‐bacterial properties of these functional nanosheets in vitro. Finally, the in vivo therapeutic effects of aptamer‐modified nanosheets on otitis media were evaluated in the ovalbumin (OVA)‐induced OME rat model.

## 2 Results and Discussion

### 2.1 The Correlation Between NET/EET Levels and the Severity of Inflammation

**Figure 1:**
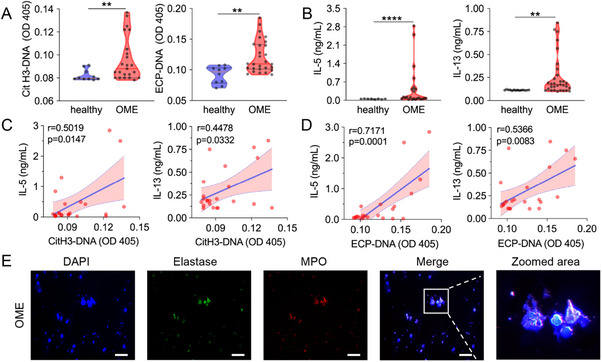
The correlation between NETs/EETs and the severity of OME patients. A) The levels of CitH3‐DNA and ECP‐DNA complexes in MEE from OME patients and plasma from healthy volunteers. Data are presented as the mean ± SD. Differences were assessed by the student‐*t*‐test of variance (^*^ *p* < 0.05, ^**^ *p* < 0.01, ^***^ *p* < 0.001). C) The correlation between the CitH3‐DNA and IL‐5, IL‐13 levels in MEE from OME patients. D) The correlation between the ECP‐DNA and IL‐5, IL‐13 levels in MEE from OME patients. The correlation of clinical data was assessed by the Spearman correlation coefficient (r). E) Representative immunofluorescence images of DAPI, Elastase, and MPO staining of MEE smears. Scale bar, 200 µm.

To investigate the NETs, EETs, dsDNA, and miRNA in middle ear effusion (MEE) of patients with OME, we collected 31 MEE samples from OME patients. To detect the presence of NETs and EETs in the MEE of OME patients, both immunofluorescence imaging and composite enzyme‐linked immunosorbent assay (ELISA) techniques were employed.^[^ ^22^ ^]^ In addition, the levels of pro‐inflammatory cytokines were measured using the corresponding ELISA kits, including interleukin‐6 (IL‐6), IL‐5, IL‐13, and IL‐1β, to assess the inflammatory severity in the middle ear. The relative concentrations of dsDNA and miRNA in the MME or plasma were also determined using the pico‐green or ribo‐green kits. The results showed that citrullinated histone H3‐DNA (CitH3‐DNA), eosinophil cationic protein‐DNA (ECP‐DNA), dsDNA, and miRNA levels were significantly higher in the MEE of OME patients than in healthy volunteers (**Figure** **1**A; Figure S1A, Supporting Information). Furthermore, the levels of pro‐inflammatory cytokines, including IL‐5, IL‐13, IL‐6, and IL‐1β, in the MEE of OME patients were also considerably elevated compared to healthy volunteers (Figure 1B; Figure S1B, Supporting Information), proving an abnormal inflammatory status in the middle ears of OME patients.

As a double‐edged sword, ETs not only play a beneficial role in innate immune defense against pathogen infection but also cause tissue damage and inflammatory responses through their enzymes, active proteins, and DNA structures.^[^ ^23^ ^]^ In our study, a strong positive correlation was then observed between CitH3‐DNA and ECP‐DNA and several inflammatory cytokines (IL‐5, IL‐13, IL‐6, and IL‐1β) in MEE of OME patients (Figure 1C,D; Figure S2, Supporting Information), indicating a definite association between ETs and the inflammatory response. Considering that the compositions of NETs/EETs are primarily a dsDNA and histone framework, and dsDNA can serve as biological markers of NETs/EETs,^[^ ^24^ ^]^ we further investigated the correlation between dsDNA levels and these inflammatory factors. As shown in Figure S3A,B (Supporting Information), obvious positive correlations were also observed between dsDNA and these inflammatory cytokines in MEE, proclaiming a tight relation between dsDNA and the inflammatory response in OME patients. A similar positive correlation was also observed between miRNA and IL‐6 and IL‐1β (Figure S3C, Supporting Information). Immunofluorescence staining also demonstrated fibrous structures of extracellular dsDNA co‐localizing with both elastase and myeloperoxidase (MPO), suggesting the overdone existence of NETs in the MEE of OME patients (Figure 1E). Meanwhile, extracellular dsDNA co‐localizing with ECP, as the indicator of EETs, was also observed in the MEE of OME patients (Figure S4, Supporting Information). All these data revealed the elevation of NETs and EETs in the middle ears of OME patients, highlighting the critical role of NETs/EETs in the dysregulated inflammatory response. The above data collectively unraveled that NETs/EETs played a crucial role in regulating pathological processes such as inflammatory response, hyperresponsiveness, and mucus secretion in the middle ear, indicating the potential to parse out targeted strategies for better therapeutic outcomes.

### 2.2 The Synthesis and Characterization of Aptamer‐Modified Nanosheets

**Figure 2:**
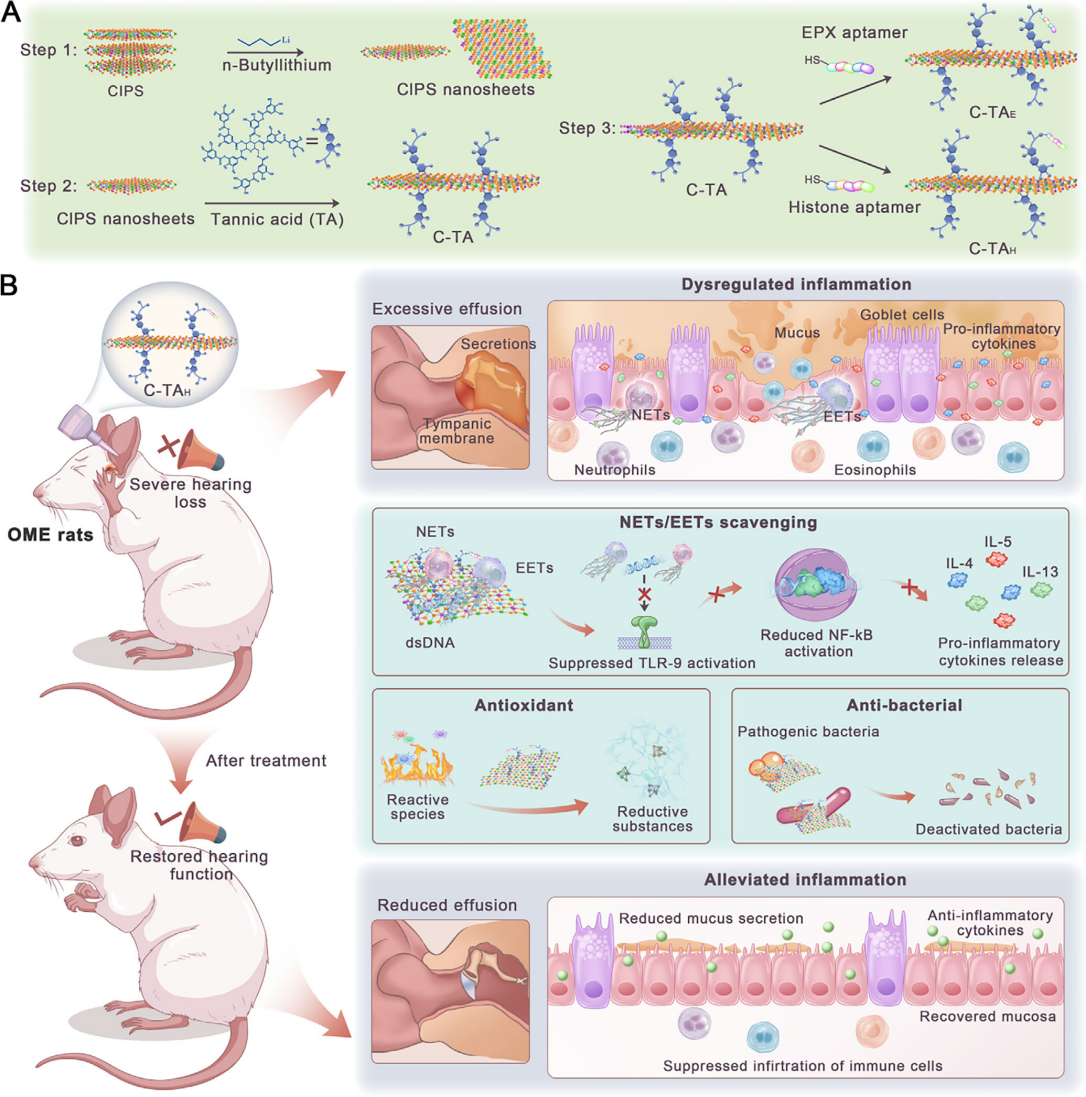
Schematic illustration of multifunctional nanosheet preparation and the application for OME treatment. A) Schematic of the fabrication of C‐TA_H_. B) The intervention of the inflammatory cascade in an OME rat model with C‐TA_H_. NETs, neutrophil extracellular traps; EETs, eosinophilic extracellular traps; CIPS, CuInP2S6; TA, tannic acid; C‐TA_H_, CIPS modified by TA and histone aptamers.

The above experiments disclosed that elevated NET/EET levels in OME patients were probably associated with excessive inflammation in the middle ear. Previous research also reported that ETs might contribute to the recurrence and chronicity of the disease, as well as the clogging of tympanostomy tubes in children with OME.^[^ ^25^ ^]^ Consequently, ETs‐targeted strategies might serve as an inspiring treatment for OME. While DNase I‐mediated degradation of NETs/EETs has demonstrated therapeutic benefits in relieving severe airway inflammatory diseases, considerations regarding its stability, cost, and potential side effects continue to warrant attention.^[^ ^26^ ^]^ Fortunately, the results from our research team and others unveiled the extracellular dsDNA scavenging strategies with nanomaterials displayed excellent therapeutic efficacy for several intractable inflammatory diseases, such as sepsis, trauma, chronic rhinosinusitis (CRS), and diabetic wound healing.^[^ ^27^ ^]^ However, the importance of ETs‐targeted strategies was unfortunately neglected in these studies, retarding the further improvement of therapeutic efficacy. dsDNA could be adsorbed onto the cationic nanomaterials via electrostatic interactions, while the NETs/EETs binding efficacies were attenuated due to the intricate composition.^[^ ^14a^ ^]^ Therefore, we developed aptamer‐modified nanosheets for the targeted strategies of ETs in the inflammatory tissues of OME patients (**Figure** **2**A). We speculated that such an ET targeted strategy was promising to be effective in OME treatment, which has been barely explored in previous publications (Figure 2B).

**Figure 3:**
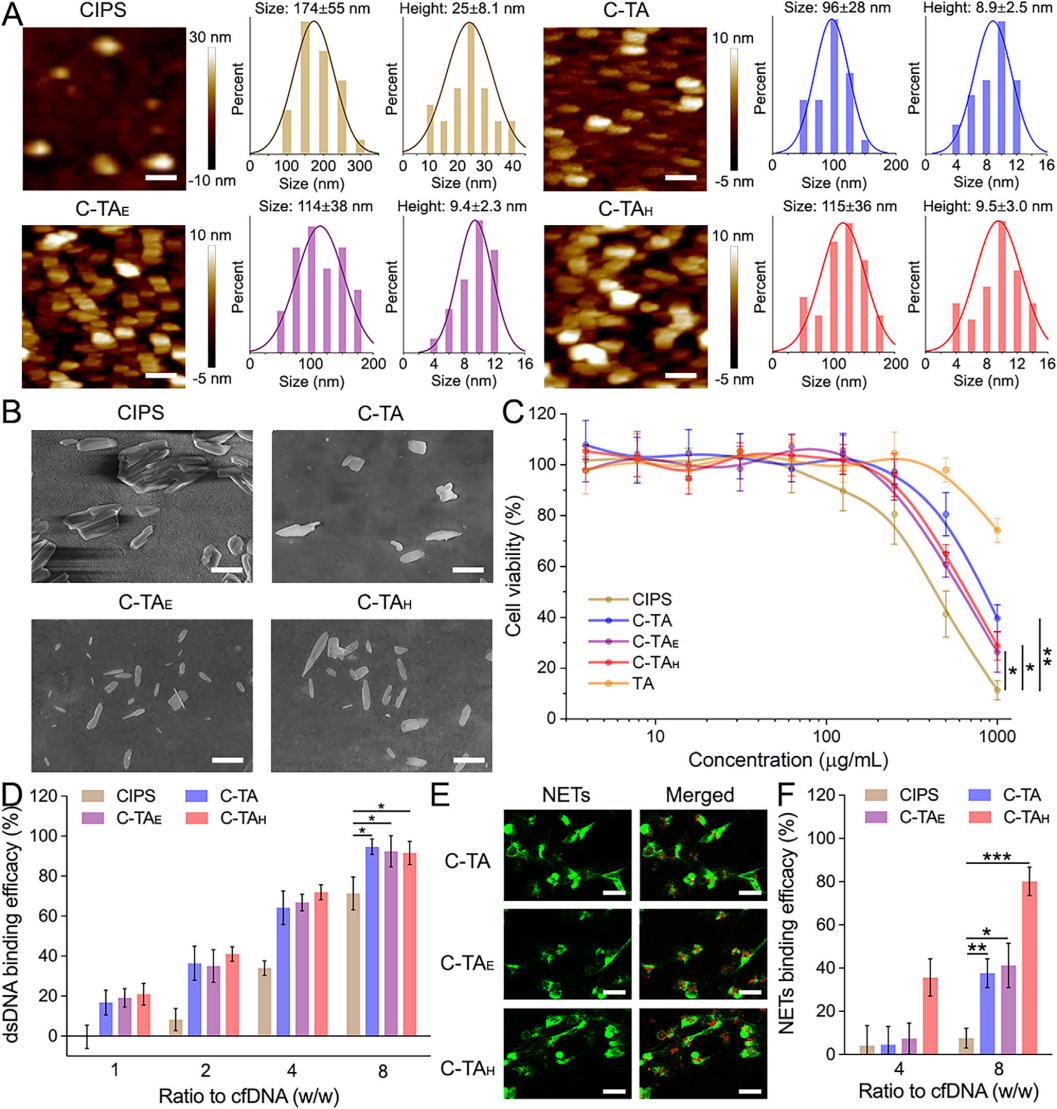
Synthesis and characterization of multifunctional nanosheets. A) AFM images and corresponding size and height distributions of CIPS, C‐TA, C‐TA_E_, and C‐TA_H_. Scale bars: 200 nm. B) SEM images of CIPS, C‐TA, C‐TA_E_, and C‐TA_H_. Scale bars: 200 nm. C) Cell viability in different groups treated with CIPS, C‐TA, C‐TA_E_, and C‐TA_H_ for 24 h. D) Quantitative dsDNA binding capacities of CIPS, C‐TA, C‐TA_E_, and C‐TA_H_, assessed using the Picogreen assay. E) The CLSM images of NETs incubated with Cy5‐labeled C‐TA, C‐TA_E_, and C‐TA_H_ in PBS. Scale bars: 10 µm. F) Quantitative NETs binding capacities of CIPS, C‐TA, C‐TA_E_, and C‐TA_H_, assessed using the fluorescence co‐localization analysis. Data are presented as mean ± SD (One‐way ANOVA, ^*^ *p* < 0.05, ^**^ *p* < 0.01, ^***^ *p* < 0.001).

In this study, we prepared CIPS nanosheets (NSs) through exfoliation of bulk CIPS with the Li‐ion intercalation protocol (Figure S5, Supporting Information).^[^ ^21^ ^]^ CIPS was chosen as an ultrathin 2D backbone, which is attributed to its outstanding broad‐spectrum anti‐virus and anti‐bacterial efficacy, stability for long‐term storage, and remarkable biocompatibility.^[^ ^21^ ^]^ The nuclear magnetic resonance (NMR), Fourier transform infrared spectrometer (FTIR), UV–vis absorption, and Elemental Mapping Data proved the successful synthesis of C‐TA, eosinophil peroxidase (EPX) aptamer‐modified C‐TA (C‐TA_E_) and C‐TA_H_ (Figures S6–S10, Supporting Information). The exfoliated CIPS exhibited a typical 2D sheet‐like morphology and a few‐layered structure with a mean size of 174 ± 55 nm and a mean height of 25.0 ± 8.1 nm, determined by atomic force microscopy (AFM) (**Figure** **3**A). In contrast, the size and height of nanosheets after reaction with TA decreased to 96 ± 28 and 8.9 ± 2.5 nm, respectively, indicating TA modification also facilitated the full exfoliation of CIPS. TA, with its abundant phenolic hydroxyl groups, forms strong hydrogen bonds and coordination complexes with surface metal ions in CIPS. These interactions effectively weaken interlayer van der Waals forces while simultaneously facilitating exfoliation.^[^ ^28^ ^]^ In the next step, EPX and histone aptamers were conjugated to fabricate aptamer‐modified C‐TA (C‐TA_E_ and C‐TA_H_) to achieve targeted ET binding. The sizes and heights of C‐TA_E_ (114 ± 38 and 9.4 ± 2.3 nm) and C‐TA_H_ (115 ± 36 and 9.5 ± 3.0 nm) were a little larger than C‐TA, substantiating that aptamer modification did not significantly change the nanostructure of 2D nanosheets. The modification ratios were ≈35% for C‐TA_H_ and 44% for C‐TA_E_ (Figure S9, Supporting Information). TA and aptamers were successfully grafted onto C‐TA, which was further confirmed by the Elemental Mapping Data of C‐TA_H_ obtained via Energy Dispersive Spectroscopy (EDS); Cu and In are unique to CIPS, N is specific to the aptamer, while C and O are shared by tannic acid and the aptamer (Figure S10, Supporting Information). The sizes of functional nanosheets were also confirmed by the scanning electron microscopy (SEM) and dynamic light scattering (DLS) data (Figure 3B; Figure S11, Supporting Information), and the zeta potential of all nanosheets was negatively charged, owning to the TA coverage. Interestingly, C‐TA, C‐TA_E_, and C‐TA_H_ exhibited markedly lower cytotoxicities than bare CIPS nanosheets, proving the biocompatibility of TA coverage (Figure 3C; Figure S12, Supporting Information). This difference is particularly pronounced at higher concentrations and extended exposure durations. We attribute this behavior to the unmodified surface of bare CIPS, which may promote nonspecific cellular interactions and enhanced uptake, thereby exacerbating cytotoxic effects.

### 2.3 The NETs/EETs Binding and Anti‐Inflammation of Multifunctional Nanosheets

As shown in Figure 3D, the dsDNA binding efficiency of C‐TA, C‐TA_E_, and C‐TA_H_ was higher than that of CIPS, determined by the pico‐green assay. Most previous dsDNA scavengers were based on the electrostatic interaction between cationic nanomaterials and anionic base pairs in DNA molecules.^[^ ^27^, ^29^ ^]^ Nevertheless, negatively charged C‐TA also manifested robust dsDNA binding affinity, attributed to the hydrogen bonding between TA and the phosphate backbone of DNA molecules.^[^ ^17^ ^]^ In view of the previous studies, the NETs/EET binding by nanomaterials is extremely difficult compared to dsDNA alone due to the relatively large volume and complicated composition.^[^ ^2^, ^14^ ^]^ Aptamers, as short, single‐stranded nucleic acids (DNA or RNA), have been engineered to bind to targeted proteins with high affinity specifically.^[^ ^19^ ^]^ In this work, EPX and histone aptamers were modified onto nanosheets (C‐TA_E_ and C‐TA_H_) to prepare targeted NETs/EET scavengers, respectively. Although C‐TA, C‐TA_E_, and C‐TA_H_ displayed similar dsDNA binding efficacy, significant differences were detected for the NETs/EETs binding tests between the nanosheets with or without aptamers. In consistence, fluorescence colocalization between NETs and the nanosheets was observed with varying degrees, with a notable increase in binding efficiency in the C‐TA_H_ group (≈80%) compared to the C‐TA (≈38%) and C‐TA_E_ groups (≈41%) at 8 µg mL^−1^ (Figures 3E,F). C‐TA_H_ also exhibited much more potent EET binding efficiency (Figure S13, Supporting Information), suggesting that the C‐TA_H_ nanosheets displayed robust binding affinity to NETs/EETs due to the coating of specific aptamers. EPX is an eosinophil‐specific protein recognized for its potent oxidizing and antibacterial properties.^[^ ^4^ ^]^ Normally localized within eosinophil granules, EPX contributes to immune responses and bactericidal activity. However, EPX content in EETs is relatively low compared to histones, as EPX primarily functions as an active enzyme that becomes incorporated into the DNA network.^[^ ^5^ ^]^ In contrast to EPX, histones serve as fundamental structural components of EETs, binding to DNA to form stable mesh‐like structures.^[^ ^6^ ^]^ Histones are present in greater abundance within EETs, especially throughout the DNA backbone.^[^ ^7^ ^]^ Consequently, simultaneously targeting both histones and cfDNA yields stronger binding and more effective outcomes than targeting EPX and cfDNA together. Upon binding to ETs, C‐TA_H_ effectively blocks the activation of PRRs on immune cells and the subsequent release of inflammatory cytokines. In addition, the isolated NETs were added to Raw 264.7 cells, and the mRNA expressions of IL‐1β, IL‐6, and TNF‐α were promoted after NETs incubation based on the results of quantitative real‐time polymerase chain reaction (qRT‐PCR) (Figure S14, Supporting Information). As expected, C‐TA_H_ suppressed the NETs‐elicited release of pro‐inflammatory cytokines from Raw264.7 cells, confirming the strong NETs binding capacity.

Antimicrobial treatment is important for controlling bacterial infection and preventing chronic inflammation of otitis media.^[^ ^30^ ^]^ The application of antibiotics in the local middle ear cavity for OME treatment is seriously restricted by the increasing frequency of multidrug resistance (MDR).^[^ ^31^ ^]^ Therefore, the combination of anti‐inflammation and antibacterial should be taken into consideration when developing functional nanoplatforms for more efficient treatment of OME patients. Fascinatingly, some transition metal disulfides, such as MoS_2_ and WS_2_, have been proclaimed to hold antibacterial activity, typically through physically piercing cell membranes of bacteria like a sharp knife and generating reactive oxygen species (ROS).^[^ ^32^ ^]^ CIPS was selected in this work because of its unique physical and chemical properties as well as excellent biocompatibility.^[^ ^33^ ^]^ Given the 2D layered sheet‐like nanostructure, CIPS and its derivatives probably possess potent antibacterial properties. As expected, C‐TA, C‐TA_E_, and C‐TA_H_, considerably retarded the growth of both Gram‐negative (*E. coli*) and Gram‐positive (*S. aureus*) bacterial lines (Figure S15, Supporting Information). The growth curve, quantified by optical density at 620 nm (OD620) from the bacteria supernatant, also showed that CIPS, C‐TA, C‐TA_E_, and C‐TA_H_ possessed evident antibacterial activity against both *E. coli* and *S. aureus* (Figure S16, Supporting Information). Of note, TA and aptamer modifications of nanosheets did not markedly reduce the antibacterial effect of CIPS.

Excessive ROS generated by injured cells is another mechanism that significantly accelerates the progression of otitis media.^[^ ^34^ ^]^ Elevated ROS levels can cause DNA damage, leading to loss of cell function and even cell death, which perpetuates inflammation and contributes to the progression of chronic otitis media.^[^ ^34^, ^35^ ^]^ In consequence, ROS reduction by functional nanosheets was calculated by determining intracellular ROS levels using cell‐permeant dichlorodihydro fluorescein diacetate (DCFH‐DA). The introduction of C‐TA, C‐TA_E_, and C‐TA_H_ decreased the fluorescence intensity in LPS‐treated cells, indicating effective ROS scavenging by the nanosheets in vitro (Figure S17, Supporting Information). These data jointly demonstrated the potential application of multifunctional C‐TA_H_ for OME treatment through ETs targeted binding, antibacterial, and antioxidant properties.

### 2.4 C‐TA Reversed the Hearing Efficacy in an OME Rat Model

**Figure 4:**
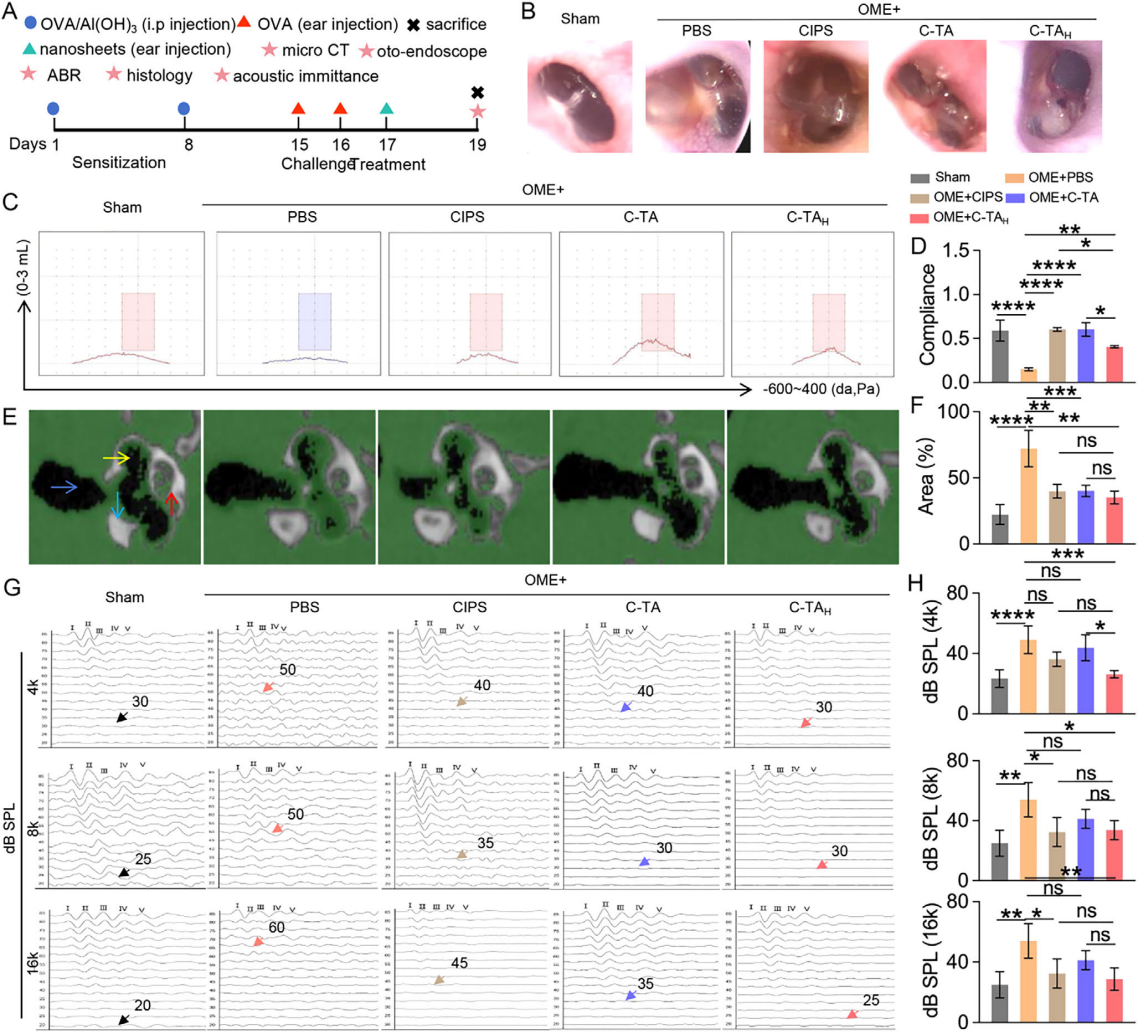
C‐TA_H_ restored hearing functions in the OVA‐induced OME rat model. A) The schematic diagram of the establishment of the OME rats with different treatments. B) The morphologies of the tympanic membranes of experimental rats, viewed through an endoscope. C) The acoustic immittance of rats in different groups. D) Statistical analysis of acoustic immittance results. E) micro‐CT images of ears of experimental rats (The red arrow indicates the cochlea, the yellow indicates the middle ear cavity, the blue indicates the mastoid, and the green indicates the external auditory canal). F) Statistical analysis of the volume of exudate in the middle ear based on the micro‐CT results. G) Auditory brainstem response (ABR) levels and threshold value. F) Statistical analysis of threshold value according to the ABR tests. Data are presented as mean ± SD (One‐way ANOVA, ns represents no significance, ^*^ *p* < 0.05, ^**^ *p* < 0.01, ^***^ *p* < 0.001, and ^****^ *p* < 0.0001).

Building upon the findings that C‐TA_H_ exhibited superior targeted binding for dsDNA and ETs, ROS scavenging, and anti‐bacterial efficiency in vitro, we conducted an in vivo assessment of the therapeutic efficacy using an OVA‐induced OME rat model.^[^ ^36^ ^]^ The animal model was established by administering initial systemic sensitization through intraperitoneal injection of OVA on the 1st and 8th day, followed by a challenge with an intra‐aural injection of OVA on the 15th day and the 16th day (**Figure** **4**A). The rats treated with PBS at the corresponding time points served as a sham group. On the 17th day, the rats in the different treatment groups were anesthetized and then respectively injected with PBS, CIPS, C‐TA, and C‐TA_H_ (0.5 µg in 20 µL PBS) into the bilateral middle ear cavity.

We first assessed the biodistribution and accumulation of these nanosheets (labeled by Cy5) in the ear tissues of OME model rats. The fluorescent signals in the middle ear gradually declined after injection and were completely undetectable by day 7, demonstrating efficient clearance from the body and underscoring the high safety profile of the nanomaterials. Importantly, the materials persisted in the middle ear for at least 3 days, ensuring sufficient duration to exert their therapeutic effects (Figure S18, Supporting Information). This limited retention minimizes the risk of systemic exposure and potential unintended immunosuppressive effects. Additionally, no detectable fluorescence accumulated in the heart, liver, spleen, lungs, kidneys, or brain, indicating negligible systemic distribution and suggesting a low likelihood of organ toxicity or neurotoxicity with this administration route (Figure S19, Supporting Information). These findings reinforce the safety of the localized delivery strategy employed in our study.

The therapeutic effects in different treatment groups were determined with an oto‐endoscope, auditory brainstem response (ABR) threshold, micro‐computed tomography (micro‐CT), histochemical analysis, and acoustic immittance measurements. As depicted in Figure 4B, the tympanic membrane in the Sham group was clear with no secretion in the tympanic cavity, while otopiesis and effusion were observed in the OME+PBS group. Additionally, the ear canal in PBS‐treated OME rats exhibited clear radial vascularity, suggesting a sustained inflammatory response. Fortunately, a semi‐transparent tympanic membrane without evident vascular proliferation was discovered in the C‐TA_H_ group, and the therapeutic effects outperformed that in OME rats treated with CIPS and C‐TA.

In addition to the organic damage, hearing loss is another feature of OME patients.^[^ ^37^ ^]^ The typical type A acoustic immittance diagram of the rats in the Sham group was observed. In contrast, those in the OME+PBS group showed type B diagrams with no evident peak sound compliance, demonstrating the accumulation of MEE in the middle ear cavity restrained sound conduction (Figure 4C,D). Consistent with previous results, the acoustic immittance diagram of the rats in the C‐TA_H_ treatment group recovers to the type A diagrams with typical peak sound compliance. Furthermore, the mucus secretion in the middle ear cavity in rats across different treatment groups was evaluated using micro‐CT testing. As depicted in Figure 4E,F, a large amount of secreted mucus occupied the middle ear cavity of the rats in the OME group (72.1 ± 13.8% area), while very few soft tissue densities shadows were found in the sham rats (22.3 ± 7.5% area). At the same time, C‐TA_H_ treatment could largely reduce the middle ear secretion (35.2 ± 4.7% area). Additionally, the C‐TA_H_ group displayed better therapeutic effects than CIPS and C‐TA‐treated OME rats in all these experiments, indicating the crucial roles of TA and aptamer modification in nanomedicine development for OME treatment.

In the pathological process of otitis media, the excessive effusion enters the middle ear cavity and then drives mechanical interference with the acoustic inputs to the inner, resulting in conductive hearing loss (CHL).^[^ ^38^ ^]^ This condition, often associated with the middle ear, can manifest as varying fluid viscosity (serous or mucous) and volume, potentially leading to a conduction loss of 30 dB or more.^[^ ^38b^ ^]^ In the current study, conspicuous elevation of ABR threshold was observed in the rats in the OME group compared to PBS‐injected sham rats in the frequency of 4 kHz (40–65 vs 20–30 dB), 8 kHz (40–65 vs 20–35 dB), 16 kHz (45–80 vs 20–30 dB), 24 kHz (45–75 vs 30–35 dB), to 32 kHz (40–70 vs 20–35 dB). Notably, rats in the OME+C‐TA_H_ group had extremely decreased ABR thresholds in each frequency (Figure 4G,H; Figure S20, Supporting Information), for instance, 4 kHz (40–65 to 25–35 dB), 8 kHz (40–65 to 25–40 dB), and 16 kHz (45–80 to 25–35 dB). In addition, the decline was more significant than that in CIPS and C‐TA‐treated rats, confirming that C‐TA_H_ treatment reduced the overall severity and improved hearing in OME rats. Furthermore, we assessed long‐term hearing recovery at day 9 post‐treatment and compared the therapeutic efficacy of C‐TA_H_ with DNase I (Figures S21 and S22, Supporting Information). While DNase I partially improved hearing in OME rats, the changes did not reach statistical significance. In contrast, C‐TA_H_ demonstrated rapid and robust improvements, enhancing low‐frequency hearing (4, 8, and 16 kHz) as early as day 2. By day 9, significant hearing recovery extended across a wider frequency spectrum, including 4, 8, 16, 24, and 32 kHz. Complementing these functional improvements, micro‐CT imaging revealed that C‐TA_H_ more effectively cleared mucous effusion from the middle ear cavity in OME mice compared to controls (Figure S23, Supporting Information). Collectively, these findings indicate that C‐TA_H_ not only promotes sustained hearing recovery in OME rats but also exhibits superior therapeutic efficacy over DNase I, underscoring its clinical potential.

### 2.5 C‐TA_H_ Modulated the Dysregulated Inflammation in the Rat Model

**Figure 5:**
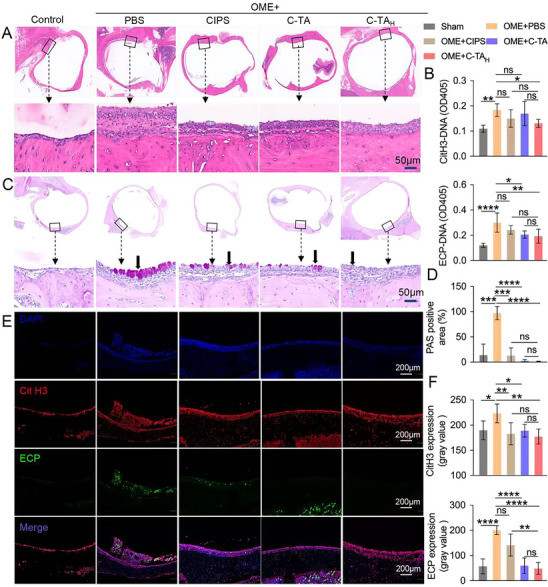
C‐TA_H_ alleviated inflammation and NETs/EETs formation in the middle ears of OME rats. A) H&E staining of the middle ear from OME rats in different treatment groups. B) The relative CitH3‐DNA and ECP‐DNA levels in the middle ear lavage fluid from experimental rats. C) PAS staining results of middle ear tissues from OME rats in different treatment groups. The goblet cells (indicated by arrows) and mucin secretions were stained purple‐red. D) The quantitative PAS‐positive area in the PAS staining slices of experimental rats. E) Representative immunofluorescence images of CitH3, ECP, and DAPI staining of middle ear tissues from OME rats in different treatment groups. F) The relative mean fluorescence intensity of CitH3 and ECP was calculated using Image J software. Data are presented as mean ± SD (One‐way ANOVA, ns represents not significant, ^*^ *p* < 0.05, ^**^ *p* < 0.01, ^***^ *p* < 0.00, and ^****^ *p* < 0.0001).

The histological analysis involved hematoxylin‐eosin (H&E), periodic acid‐Schiff (PAS) staining, and CitH3, ECP, and MPO immunostaining was exploited to assess the inflammatory status and NET/EET formation in middle ear tissue. Compared to the sham rats, the OME group exhibited noticeable mucosa thickening, which is attributed to the overdone edema and immune cell infiltration in inflammatory tissues (**Figure** **5**A). In addition, the number of cells was also calculated to be higher in the middle ear lavage fluid of OME rats than in the sham group (Figure S24, Supporting Information). Fascinatingly, C‐TA_H_ treatment mitigated the thickened mucosa and exudated cells in the lavage fluid, indicating an anti‐inflammation effect. Meanwhile, the relative levels of dsDNA, CitH3‐DNA, and ECP‐DNA were also considerably elevated in the middle ear lavage fluid of OME rats (Figure 5B; Figures S25 and S26, Supporting Information). Consistent with the in vitro results, C‐TA_H_ treatment potently reduced the elevated levels of dsDNA (5.8 ± 2.3 to 2.8 ± 0.8 µg mL^−1^), CitH3‐DNA (OD405: 0.18 ± 0.02 to 0.13 ± 0.02), and ECP‐DNA (OD405: 0.30 ± 0.08 to 0.19 ± 0.06) in the middle ear lavage fluid of OME rats, and the efficacy was more effective than the CISP and C‐TA treatment groups. Goblet cell hyperplasia, which induces over much mucus secretion in the inflammatory tissues, is another characteristic of OME;^[^ ^39^ ^]^ hence, PAS staining was utilized to observe the goblet cells in the middle ear tissues of experimental rats. Pronounced goblet cell hyperplasia was observed in the middle ear of the OME model rats, and the hyperplasia was significantly alleviated after the treatment with functional nanosheets (Figure 5C,D). Moreover, it was revealed in immunofluorescence staining images that NETs and EETs were obviously elevated both in the lavage fluid and middle ear mucus in OME rats compared to the sham group (Figure 5E,F; Figures S27–S29, Supporting Information). At the same time, C‐TA_H_ treatment markedly reduced the levels of NETs (by 21%) and EETs (by 76%) in inflammatory tissues, and the suppression is more effective than the CISP and C‐TA treatment groups.

**Figure 6:**
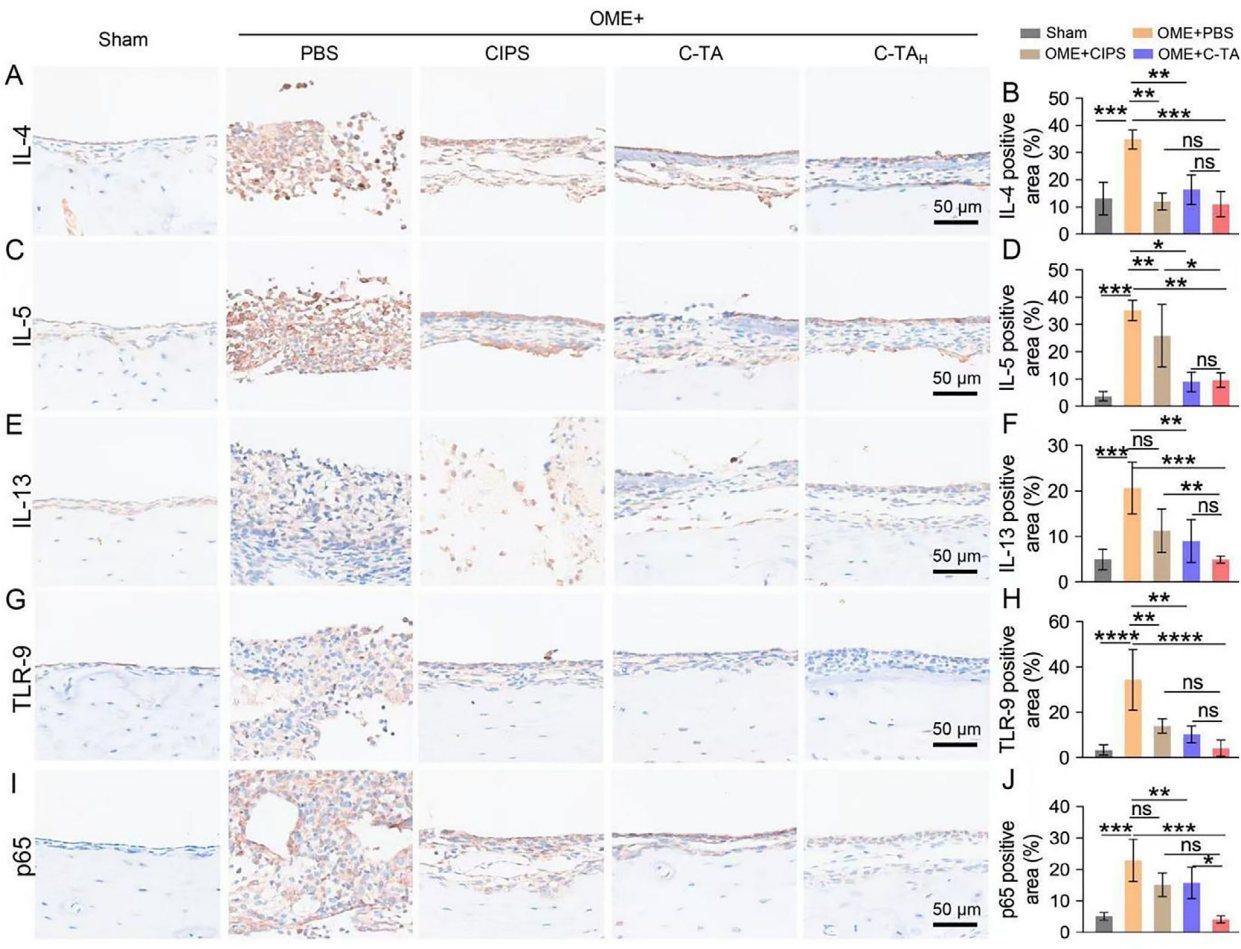
C‐TA_H_ attenuated the inflammatory response in the middle ears of the OME model rats. Representative immunohistochemical staining images and quantitative assessment of A, B) IL‐4, C, D) IL‐5, E, F) IL‐13, G, H) TLR 9, and I, J) p65 in middle ears from OME rats in different treatment groups. Data are presented as mean ± SD (One‐way ANOVA, ns represents no significance, ^*^ *p* < 0.05, ^**^ *p* < 0.01, and ^***^ *p* < 0.001).

Sensitized neutrophils and eosinophils can generate NETs and EETs, which are capable of killing bacteria and phagocytosing necrotic cells and allergens.^[^ ^40^ ^]^ However, over much NET/EET formation can damage healthy tissues of the middle ear, followed by the recruitment of immune cells, resulting in the enhanced secretion of pro‐inflammatory mediators.^[^ ^41^ ^]^ As shown in **Figure** **6**A–F, elevated levels of pro‐inflammatory cytokines, including IL‐4, IL‐5, and IL‐13, were observed in the mucous membrane of the middle ear of OME rats compared to the sham group. C‐TA_H_ treatment greatly declined the levels of IL‐4 (34.9 ± 3.5% to 11.0 ± 4.6%), IL‐5 (34.7 ± 5.1% to 9.6 ± 2.7%), and IL‐13 (20.6 ± 5.6% to 4.9 ± 0.8%), which is nearly equal to healthy levels. In addition, C‐TA_H_ displayed noticeably higher anti‐inflammation efficacy than CISP and C‐TA treatments. Both dsDNA and NETs/EETs were reported to activate Toll‐like receptor 9 (TLR‐9), and the following phosphorylate nuclear factor kappa‐B (NF‐κB), initiating the uncontrolled inflammatory response.^[^ ^42^ ^]^ Similar to pro‐inflammatory cytokines, the expression of TLR‐9 and p65 (critical protein in NF‐κB pathway) were elevated ≈10‐ and 5‐fold in the OVA‐induced OME group. As expected, C‐TA_H_ treatment extremely decreased both TLR‐9 and p65 proteins to almost normal levels, which is also more effective than CIPS and C‐TA treatments (Figure 6G–J). Taken together, all these outcomes demonstrated that C‐TA_H_ could effectively suppress the excessive NET/EET formation, dysregulated inflammatory response, and overdone mucus secretion in the middle ear cavity of OVA‐treated rats. More importantly, the hearing loss of the OME rats was successfully reversed after C‐TA_H_ treatment in all examined frequencies (from 4 to 32k Hz). In addition, our findings manifested the pivotal roles of TA and aptamer modification in constructing therapeutic nanoplatforms with robust NETs/EETs elimination, ROS reduction, and antibacterial properties.

### 2.6 Transcriptome Analysis of the Therapeutic Results

**Figure 7:**
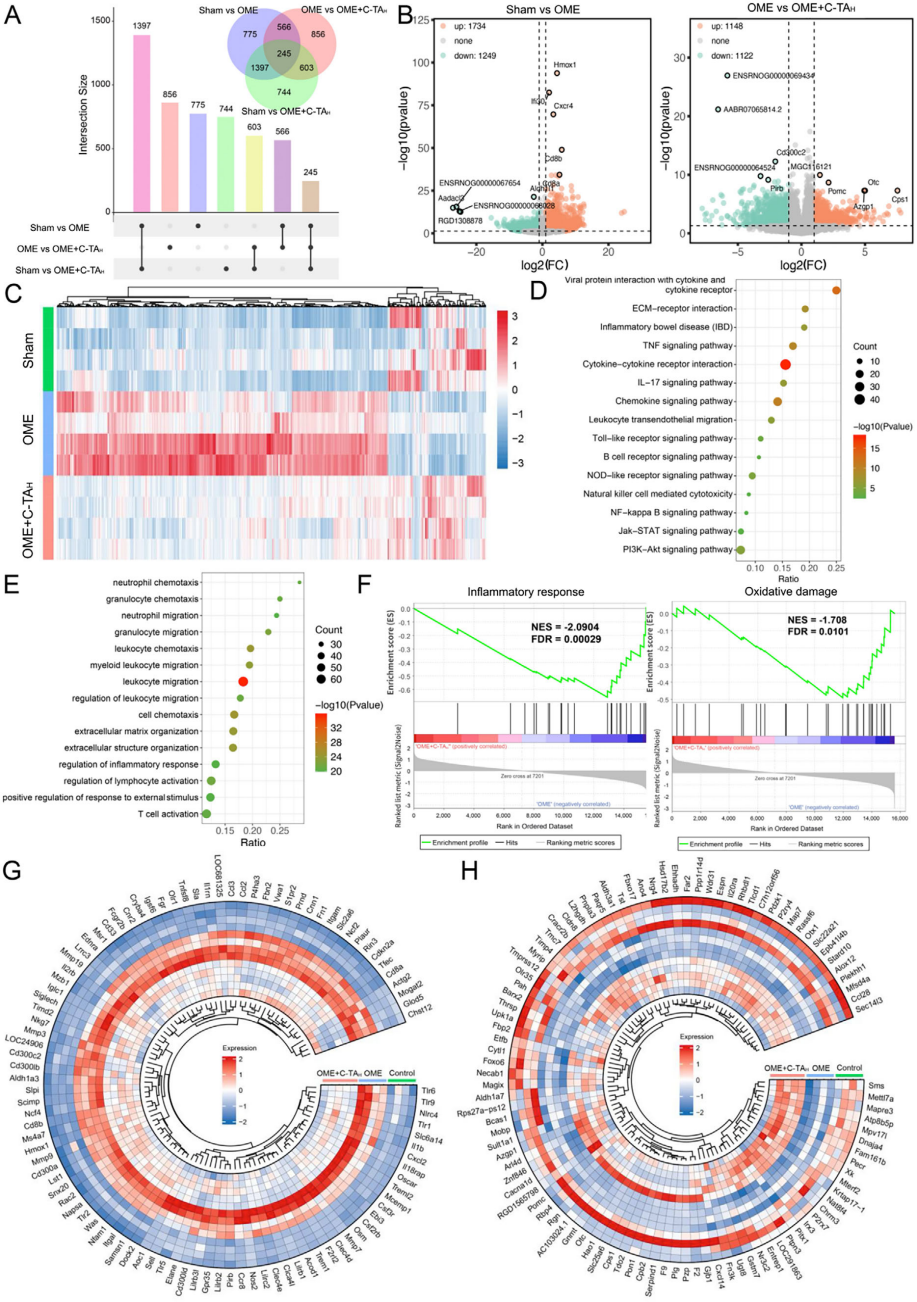
Transcriptome analysis based on RNA sequencing. A) UpSet plot and Venn map of DEGs between the Sham group, OME group, and OME+C‐TA_H_ group. B) Volcano maps based on the Sham versus OME group and OME versus OME+C‐TA_H_ group. C) Heat map of the DEGs with significant changes between the experimental groups. D) KEGG pathway analysis of DEGs in the 626 genes that were upregulated in the OME group compared to the Sham group and subsequently downregulated in the OME+C‐TA_H_ group. E) GO term enrichment analyses of these 626 DEGs. F) GSEA results based on the KEGG gene set of these 626 DEGs. The dotted line represents the zero line. G) Heat maps of overlapped genes that were upregulated in the OME versus Sham comparison, and then down‐regulated in the OME+C‐TA_H_ versus OME group. H) Heat maps of overlapped genes that were downregulated in the OME versus Sham comparison and then down‐regulated in the OME+C‐TA_H_ versus OME group.

To comprehensively evaluate the anti‐inflammation effect of C‐TA_H_ for OME, transcriptome analysis based on RNA‐sequencing was conducted for middle ear tissues from three experimental groups: the Sham group, the OME group, and the OME+C‐TA_H_ group. The Veen map uncovered 2983 and 2270 differentially expressed genes (DEGs) in the comparisons of Sham versus OME and OME versus OME+C‐TA_H_, respectively, with 801 overlapping genes (**Figure** **7**A,B). Further analysis of the volcano plots showed that 1734 upregulated genes and 1249 downregulated genes were found in the OME group compared to the Sham rats. In contrast, there were 1148 upregulated genes and 1122 downregulated genes in the OME+C‐TA_H_ group against the OME rats (Figure 7B). The heatmap analysis depicted that the variations of DEGs in Sham versus OME rats were mostly reversed after C‐TA_H_ treatment, supporting the finding that C‐TA_H_ mitigated the OVA‐stimulated inflammation in the middle ear (Figure 7C). In the next step, the 626 genes that were upregulated in the OME rats and subsequently downregulated in the OME+C‐TA_H_ group were selected (Figure S31B, Supporting Information), and Kyoto Encyclopedia of Genes and Genomes (KEGG) analysis of these genes disclosed that C‐TA_H_ treatment potently relieved several critical inflammatory pathways in OME rats. These pathways encompassed the IL‐17 signaling pathway, Toll‐like receptor signaling pathway, NOD‐like receptor signaling pathway, NF‐kappa B signaling pathway, leukocyte transendothelial migration, and B cell receptor signaling pathway, which all play crucial roles in amplifying the inflammatory response under OME conditions (Figure 7D). Additionally, Gene Ontology (GO) analysis (biological process) was also applied for the 626 DEGs, and several enriched terms were identified, including neutrophil and granulocyte chemotaxis and migration, regulation of the inflammatory response, regulation of lymphocyte activation, and T cell activation (Figure 7E). These biological processes play pivotal roles in regulating the chemotaxis and migration of inflammatory immune cells, especially in the context of OVA‐induced stress conditions.

On the contrary, the 161 genes downregulated in the OME group and subsequently upregulated in the OME+C‐TA_H_ group were also collected for KEGG and GO analysis. The analysis outcomes proclaimed that C‐TA_H_ treatment significantly modulated several important metabolic pathways, such as anti‐oxidative stress and anti‐inflammatory pathways, which were abnormally stimulated in OME conditions (Figures S32 and S33, Supporting Information). Several pathways are essential for cellular defense against oxidative stress, including the pentose phosphate pathway (PPP), beta‐alanine metabolism, glutathione metabolism, and peroxisome pathways. There are also key pathways that contribute to anti‐inflammatory effects, such as tryptophan metabolism, the PPAR signaling pathway, butanoate metabolism, and histidine metabolism. All of these pathways play critical roles in maintaining normal cell metabolism and relieving inflammation and peroxidation damage under pathological conditions of OME. The observed enrichment suggested that C‐TA_H_ administration alleviated peroxidation damage and accelerated the repair of the inflammatory pathological tissues in the OME animal model.

Gene Set Enrichment Analysis (GSEA) based on KEGG results further illuminated the therapeutic effects of C‐TA_H_. Several inflammatory pathways, including inflammatory response and oxidative damage, and the Toll‐like receptor, IL‐5, and IL‐6 signaling pathways were suppressed after C‐TA_H_ treatment (Figure 7F; Figure S34, Supporting Information). Accordingly, genes relevant to tissue damage and inflammation were chosen and comparatively examined. C‐TA_H_ mitigated the upregulation of immune response‐associated genes observed in OVA‐treated rats, such as CCL2, CCL3, IL‐1β, and TLR‐9, indicating excessive inflammation in OME was reduced (Figure 7G). Additionally, heatmaps also depicted the downregulation of epithelial function‐related genes post‐OME, which was reversed following C‐TA_H_ treatment (Figure 7H). Collectively, transcriptome analysis demonstrated that C‐TA_H_ is promising in alleviating the inflammatory response and oxidative damage of OME patients in clinical settings.

## 3 Discussion

The Prolonged overproduction of NETs/EETs can exacerbate the chronicity and severity of inflammatory disease, damaging normal tissue structures and facilitating bacterial biofilm formation.^[^ ^3^ ^]^ In OME patients, elevated levels of NETs‐ and EETs‐related proteins (e.g., major basic protein) have been identified in MEE, highlighting their role in sustaining the uncontrolled inflammatory process.^[^ ^8^ ^]^ Our findings also revealed significantly elevated levels of dsDNA, CitH3‐DNA, and ECP‐DNA in the MEE of OME patients, and positive correlations were also observed between the levels of NETs or EETs and the inflammation severity of OME patients, underscoring their potential as both diagnostic indicators and therapeutic targets. Treatment strategies targeting NETs/EETs hold promise for managing various inflammatory, autoimmune, and infectious diseases where dysregulated NET/EET formation plays a significant role. However, developing targeted therapeutic strategies to address the excessive formation of NETs/EETs remains a significant challenge. Approaches range from enzyme‐based therapies (e.g., DNase) to molecular inhibitors (e.g., PAD4 inhibitors) and immunomodulatory agents, while their clinical translation prospects are relatively low due to the high cost, low stability, and unavoided toxicities. While preclinical and clinical data are still evolving, the development of targeted therapies that regulate NET/EET formation and activity could provide significant therapeutic benefits in treating ETs‐associated diseases. However, further research is required to determine the most effective and safe strategies for clinical application.

Nanoparticles can be engineered to selectively target NETs/EETs or the components, such as histones or MPO.^[^ ^43^ ^]^ For example, nanoparticles conjugated with DNase or other therapeutic agents can be used to deliver drugs directly to areas where NETs/EETs are overproduced.^[^ ^43^, ^44^ ^]^ However, previous research has shown that the dsDNA fibers of NETs typically measure 10–15 microns in length and 15–50 nanometers in width. In local tissues, NETs/EETs can form network structures spanning ≈10–100 microns.^[^ ^45^ ^]^ As a result, conventional nanoparticles and polymers fall short in addressing the scale of micron‐sized ETs, highlighting the distinctive advantages of our 2D nanomaterial, CIPS. With its sheet‐like nanostructure, high surface‐to‐volume ratio, flexible backbone, and tunable surface chemistry, CIPS stands out as a promising candidate nanosheet, which has shown great promise in biomedical applications due to its unique properties. Our study also highlighted its potential as an antimicrobial agent, leveraging its ability to disrupt bacterial membranes and prevent biofilm formation. While initial studies suggest good biocompatibility, further in vivo investigations and surface functionalization strategies are underway to optimize its safety and efficacy for clinical applications. In this study, following modification with TA and a histone aptamer, the resulting C‐TA_H_ exhibited sizes of 115 ± 36 nm and heights of 9.5 ± 3.0 nm. The introduction of TA allowed C‐TA_H_ to form stable complexes with DNA molecules, while the incorporation of the aptamer enabled it to bind securely to the histone structures of ETs, further enhancing its functionality. Our findings show that C‐TA_H_ nanosheets displayed robust binding affinity to NETs/EETs and suppressed the NETs‐elicited release of pro‐inflammatory cytokines, while retaining its characteristic 2D nanosheet structure. Further, C‐TA_H_ exhibited excellent modulatory effects on dysregulated inflammation to ameliorate hearing loss in an OVA‐induced OME rat model. In addition, the therapeutic effects of C‐TA_H_ outperformed CIPS and C‐TA, indicating the significance of TA and histone aptamers in the construction of functional nanoscavengers. The transcriptomic analysis with RNA‐sequencing supported the potential of C‐TA_H_ as a viable therapeutic option for OME, highlighting the demand for additional preclinical investigations.

Our goal is to explore better strategies for targeting NETs/EETs structures, ultimately creating C‐TA_H_, a multifunctional nanosheet with the potential to treat ETs‐related inflammatory diseases and prevent bacterial infections and chronicity. These results reveal a previously underexplored therapeutic approach and emphasize that, beyond its known functions, CIPS, through simple surface modifications, can acquire additional functions that deserve further attention. Given the challenges currently faced by metal nanomaterials in clinical applications, further research into the clinical application forms of C‐TA_H_ is needed, especially whether C‐TA_H_ can be embedded on the surface of drainage strips during myringotomy and drainage for otitis media, rather than being directly instilled into the middle ear cavity. This would effectively avoid metal materials entering the body and further promote the clinical application of C‐TA_H_. Overall, our work proposed a new potential tackling strategy for OME patients, which also brings inspiration for the treatment of other ETs‐associated inflammatory diseases.

## 4 Experimental Section

### Clinical Samples

MEE was gathered from 31 OME patients from Eye, Ear, Nose, and Throat Hospital, Fudan University (Shanghai, China). Ten blood samples from healthy individuals were donated voluntarily by postgraduates and doctors at the Eye, Ear, Nose, and Throat Hospital. The collection of exudation and blood samples was approved by the Ethics Committee of the Eye, Ear, Nose, and Throat Hospital, Fudan University (approval number: 2023006). All patients and healthy volunteers were informed of the purpose of the donation, and the consent and signatures of consent forms were received. Blood samples were collected using EDTA anticoagulant tubes. After centrifugation at 3000 rpm for 10 min at 4 °C, the plasma was divided into sterile tubes and stored at −80 °C. The dsDNA, miRNA, IL‐5, IL‐13, IL‐1β, and IL‐6 concentrations of plasma and MEE were measured using the Quant‐iT PicoGreen dsDNA and miRNA Kit and corresponding ELISA kits, respectively, following the manufacturer's protocols.

### Establishment of OVA‐Induced OME Rat Model

All rats were cared for according to the instructions and approval by the Institutional Animal Care and Use Committee of Eye, Ear, Nose, and Throat Hospital, Fudan University (No. IACUC‐DWZX‐2024‐029). Male Sprague–Dawley rats (8 weeks old, 160–190 g) were fed and housed in an ACS animal feeding system with 12–12 h light/dark circle and free access to water and food. The OVA‐induced OME rat model was established in terms of a previous report.^[^ ^46^ ^]^ The rats were divided randomly into the following groups: the Sham group, OME+PBS group, OME+CIPS group, OME+C‐TA group, and OME+C‐TA_H_ group. During the systemic sensitization phase, experimental rats were intraperitoneally injected with 1.2 mg OVA (dissolved in 0.6 mL saline with 14 mg aluminum hydroxide as an immune adjuvant) on the 1st and 8th day after adaptive feeding. On the 15th and 16th day, rats were anesthetized with isoflurane solution and challenged with 0.1 mg OVA by injection into the bilateral middle ear cavity. The injection was through the posterior lower or anterior lower quadrant of the tympanic membrane using a micro syringe under a microscope. Rats in the sham group were sensitized and challenged exclusively with PBS. On the 17th day, the rats in the different treatment groups were anesthetized and then respectively injected with CIPS, C‐TA, and C‐TA_H_ (0.5 µg in 20 µL PBS per ear) into the bilateral middle ear cavity, while those in the sham group were injected with 20 µL PBS. On the 19th day, the acoustic immittance map and ABR thresholds were first detected as follows. Then, the experimental rats were sacrificed, and the middle ear tissues, along with the middle ear lavage fluid (rinsed with 200 µL sterile PBS), were gathered and stored at −80 °C. The middle ear tissues was fixed in 4% paraformaldehyde for subsequent H&E staining, immunohistochemical, and immunofluorescence analysis.

### Acoustic Immittance Map

Following general anesthesia with intraperitoneal pentobarbital sodium, the external ear canal of rats was disinfected with alcohol, and a probe was inserted into the ear canal to form a closed chamber. By altering the pressure in the outer ear canal, changes in eardrum activity and dynamic compliance of the middle ear were observed and recorded.

### ABR)Thresholds

The ABR thresholds were performed as previously described.^[^ ^36^ ^]^ After general anesthesia, the rats were placed on a heated mat inside a soundproof compartment. Three sub‐cutaneous needle electrodes were inserted into the ABR response of each rat: one at the apex (not inverted), one in the ipsilateral mastoid muscle (inverted), and one inserted into the muscle behind the contralateral auricle as the ground. These electrodes are connected to the Bioamp head stage (HS4 fiber, TDT). The stimulus is attenuated and filtered (low‐pass cut‐off frequency is 5 kHz). The stimulated sound was connected to the external ear canal through a 10 cm tube via an electrostatic speaker (TDT EC1), thus forming a calibrated closed system. In ABR threshold experiments, subjects receive tonal stimuli of different sound levels (5 dB intensity order) in a frequency range of 4–32 kHz to determine the threshold. The stimulation duration of each ABR was 5 msec, and the repetition rate was 21 s^−1^.

### Determination of dsDNA, CitH3‐DNA, and ECP‐DNA in Rats Middle Ear Lavage Fluid and MEE of Patients with OME

The dsDNA concentrations in the collected samples of OME rats and patients were measured using the Quant‐it PicoGreen dsDNA assay kit following the instructions. To quantify NETs in middle ear lavage fluid, an ELISA kit based on DNA‐associated CitH3 was utilized, as previously reported in the literature.^[^ ^22c^ ^]^ For the capture antibody, 100 µL of anti‐CitH3 (citrulline R2 + R8 + R17) antibody (ab5103, Abcam, 1 µg mL^−1^) was coated onto 96‐well plates and incubated overnight at 4 °C. After washing and blocking with 5% BSA, 20 µL of MEE samples and 80 µL of incubation buffer containing a peroxidase‐labeled anti‐DNA mAb (Cell Death ELISAPLUS, Roche; dilution 1:25) were added to the wells. After incubation at room temperature for 2 h, the plates were washed with PBS 3 times (300 µL each), and 100 µL peroxidase substrate (ABTS) was added. After 20 min of incubation at room temperature in the dark, 100 µL ABTS stop solution was then added, and the plates were measured at 405 nm wavelength. Finally, the relative amount of CitH3‐DNA and ECP‐DNA were calculated.

In another experiment, an ELISA kit based on DNA‐associated ECP was used to quantify EETs in samples. For the capture antibody, 96‐well plates pre‐coated with ECP from the Human RNase3/ECP (Ribonuclease A3/Eosinophil Cationic Protein) ELISA Kit (Elabscience, E‐EL‐H1379, China), and the Rat Eosinophil Cationic Protein (ECP) ELISA Kit (Cusabia, CSB‐E11238r, China) were utilized for patients and rat samples, respectively. The subsequent steps were similar to those of the above CitH3‐DNA ELISA test.

### H&E Staining

H&E staining for the tissue slices was performed as described in the previous literature.^[^ ^36^ ^]^ Simply, the fixed middle ear tissue was gradually dehydrated with ethanol and embedded in paraffin wax. The 5 µm sections were prepared on polylysine‐coated microscope adhesive slides, dewaxed with xylene, rehydrated with ethanol gradient, and stained with hematoxylin and eosin according to standard protocols and manufacturer's instructions.

### PAS Staining

The goblet cell proliferation in the middle ear mucosa of experimental rats in different treatment groups was detected using PAS staining. Simply, paraffin sections were hydrated using an alcohol gradient, then exposed to 0.5% periodic acid aqueous solution and Schiff reagent in the dark, followed by hematoxylin staining of the nuclei, and images were obtained under a microscope.

### Immunohistochemical Staining

The paraffin sections of the middle ear tympanum in different groups were dewaxed, rehydrated, and heated in a microwave oven for antigen retrieval. Subsequently, the slices were blocked with BSA and incubated with the CitH3 (ab5103, Abcam), ECP (biorbyt, United Kingdom), and MPO (Proteintech, China) primary antibody overnight at 4 °C in a humidified environment. The samples were then incubated with the corresponding secondary antibodies and then counterstained with DAPI for 5 min. The images were recorded by a microscope, and five views were randomly counted and analyzed in each group.

### Transcriptome Sequencing

RNA extraction from middle ear tissue was performed using TRIzol reagent. The samples were gathered from the Sham group (*n* = 4), OME group (*n* = 4), and OME+C‐TA_H_ group (*n* = 5). After extraction, the quality of the sample library was assessed using the Agilent 2100 Bioanalyzer. Finally, paired‐end (PE) sequencing of the libraries was conducted using Next‐Generation Sequencing (NGS) on the Illumina platform.

### Statistical Analysis

All data are expressed as Mean ± SD. Differences between two groups were analyzed using an unpaired *t*‐test, while differences among three or more groups were analyzed using one‐way ANOVA followed by Tukey's multiple comparison test. A *p*‐value of less than 0.05 was considered significant. Pearson's correlation was used to determine the association between variables. Statistical analyses were performed using GraphPad Prism 8.3.

## Conflict of Interest

The authors declare no conflict of interest.

## Supporting information



Supporting Information

## Data Availability

The data that support the findings of this study are available in the supplementary material of this article.
