# Supplementary material for: Targeting Neutrophil/Eosinophil Extracellular Traps by Aptamer‐Functionalized Nanosheets to Overcome Recalcitrant Inflammatory Disorders
Source: Adv Sci (Weinh). 2025 Jul 14;12(38):e04210. doi: 10.1002/advs.202504210 (PMC12520462; doi:10.1002/advs.202504210)
Supplement: Supplementary file 1 — Supporting Information [file ADVS-12-e04210-s001.pdf]

ADVANCED SCIENCE
Open Access

Supporting Information

for *Adv. Sci.*, DOI 10.1002/advs.202504210

Targeting Neutrophil/Eosinophil Extracellular Traps by Aptamer-Functionalized Nanosheets to Overcome Recalcitrant Inflammatory Disorders

Yongqiang Xiao, Xinyue Wang, Ming Liu, Xiao Fu, Nanfeng Zhang, Wenqing Yang, Junyi Ge, Yangyang Li, Duan Ma, Jing Ma, Weiping Wen, Dongdong Ren, Tianyu Zhang* and Zhaoxu Tu**

Supporting Information

Targeting Neutrophil/Eosinophil Extracellular Traps by Aptamer-Functionalized Nanosheets to Overcome Recalcitrant Inflammatory Disorders

Yongqiang Xiao, Xinyue Wang, Ming Liu, Xiao Fu, Nanfeng Zhang, Wenqing Yang, Junyi Ge, Yangyang Li, Duan Ma, Jing Ma, Weiping Wen, Dongdong Ren*, Tianyu Zhang*, and Zhaoxu Tu*

Y. Xiao, J. Ma, T. Zhang, X. Wang
ENT Institute, Department of Facial Plastic and Reconstructive Surgery, Eye & ENT Hospital, Fudan University, Shanghai, 200031, China.
E-mail: maj14@fudan.edu.cn

M. Liu, Z. Tu, W. Wen
Department of Otolaryngology, The Sixth Affiliated Hospital, Sun Yat-sen University, Guangzhou, Guangdong 510655, China
E-mail: tuzhx@mail.sysu.edu.cn

D. Ren, X. Fu
Department of Otology and Skull Base Surgery, EYE and ENT Hospital of Fudan University, Shanghai, 200031, China.

D. Ma, J. Ge
Key Laboratory of Metabolism and Molecular Medicine, Ministry of Education, Department of Biochemistry and Molecular Biology, School of Basic Medical Sciences, Fudan University, Shanghai, China

Y.L
Department of Endocrinology, Shuguang Hospital Affiliated to Shanghai University of Traditional Chinese Medicine, Shanghai, P.R. China;

N. Zhang, W. Yang
Department of ENT, Second Affiliated Hospital of Anhui Medical University, Hefei, Anhui, 230031, China

Keywords: dysregulated inflammation, neutrophil/eosinophil extracellular traps, copper indium thiophosphate, tannic acid, aptamers

Table of contents

1. Experimental section....3

2. Analysis of the MEE from patients with OME....10

3. Synthesis of functionalized nanosheets....14

4. NMR of functionalized nanosheets....15

5. FTIR of functionalized nanosheets....16

6. UV-vis absorption....17

7. DLS and zeta potential....19

8. Cytotoxicity....20

9. EET binding study....21

10. q-PCR tests....22

11. Anti-bacterial efficacy....23

12. Antioxidant studies....25

13. Biodistribution studies....26

14. Auditory brainstem response tests....28

15. Analysis of middle ear lavage fluid....33

16. Immunostaining slices of middle ear lavage fluid smears....35

17. Immunostaining staining of middle ear tissues....36

18. Veen maps....38

19. KEGG analysis....39

20. GO analysis....40

21. GSEA analysis based on KEGG results....41

1. Experimental section

Materials and methods. Ultra-high-purity CuInP2S6 (CIPS) powder was purchased from Beike 2D Materials Co., Ltd. (Suzhou, China). N-butyllithium in n-hexane was purchased from Shanghai Macklin Biochemical Technology Co., Ltd. (Shanghai, China). N-hexane was purchased from Beijing Inno Chem Science & Technology Co., Ltd. (Beijing, China). Tannic acid (TA), sodium hydroxide, fluorescein isothiocyanate (FITC), cell counting kit-8 (CCK-8) assay, and 4,6-diamidino-2-phenylindole (DAPI) were purchased from Fisher Scientific. Ampicillin sodium (AMP) solution was purchased from Sangon Biotech Co., Ltd. (Shanghai, China). Milli-Q water was used in all experiments. EPX aptamer (5' to 3': HS-ATGCCATCCT ACCAACGGTC GATGGATGAG TAATACAGGC CGGATGGGTA CAGTCG) and histone H4 aptamer (5' to 3': HS-AGACG TAAGT TAATT GGACT TGGTC GTGTG CGGCA CAGCG ATTGA AAT) were synthesized by Sangon Biotech Co., Ltd. (Shanghai, China).

UV-vis absorption spectra and CCK8 assays were recorded on a microplate reader (Thermo Fisher Scientific, US). Zeta potential and dynamic light scattering (DLS) data were obtained with a NanoBrook 90 Plus PALS (Brookhaven, US) in PBS pH 7.4.

Synthesis of CIPS nanosheets. The copper indium thiophosphate (CIPS) nanosheets were prepared by using lithium anions as intercalation agents as described previously.[1] In brief, CIPS powder (100 mg) was added to a 100 mL Schlenk flask, followed by the addition of 20 mL of n-butyllithium in n-hexane. Under argon, the mixture was stirred at room temperature. After 48 h, 50 mL of n-hexane was added to the reaction solution and centrifuged at 5,000 g for

10 min. The n-hexane supernatant was discarded, and the precipitate was dispersed with n-hexane and washed by centrifugation. The precipitate was dispersed in Milli-Q water and centrifuged at 10,000 g for 30 min. The supernatant was dialyzed in pure water (MWCO=3500 Da) for 48 h.

Synthesis of C-TA, C-TA$_E$, and C-TA$_H$. 10 mg CIPS nanosheets were suspended in 10 mL Milli-Q water. Then, the pH was adjusted to 8.0 using 10 M NaOH solution, and 10 mg TA was added to the above solution. Then, the mixture was stirred at room temperature for 8 h before the solution was dialyzed in pure water (MWCO=3500 K) for 48 h to obtain TA-covered CIPS (C-TA). 100 μg of aptamer (EPX aptamer or histone aptamer) was added to 10 mL of C-TA solution (1 mg/mL), and then the reaction was stirred for 8 h at room temperature. The mixture was dialyzed in pure water (MWCO=3500 K) for 48 h to obtain C-TA$_E$ or C-TA$_H$.

The conjugating efficiency of aptamers (APT). To evaluate the binding efficiency of aptamers (APT), which are DNA-based molecules, to the nanosheet formulations C-TA$_H$ and CTA$_E$, we measured aptamer concentrations before and after synthesis. Before reaction, APT and C-TA were added to the synthesis, and the pre-reaction liquid was collected (C-TA+APT); after reaction, the mixture was subjected to dialysis to remove any unbound aptamers, and the after-reaction liquid was collected (C-TA-APT). The concentration of aptamers in the pre-reaction liquid and the after-reaction liquid, and the aptamer conjugating efficiency, were calculated based on the initial and final absorption values at 260 nm.

Elemental Mapping Analysis of C-TA_H Nanosheets. Elemental mapping analysis was conducted to confirm the elemental composition and spatial distribution of the key components in the synthesized C-TA_H nanosheets. The samples were first drop-cast onto a molybdenum grid coated with a carbon film and dried under vacuum overnight. High-resolution elemental mapping was performed using a field emission transmission electron microscope (JEOLJEM-F200, e.g., JED-2300 Analysis Station) equipped with an energy-dispersive X-ray spectroscopy (EDS) system. The accelerating voltage was set to 200 kV. Prior to analysis, the microscope was calibrated using standard procedures to ensure accuracy and resolution. EDS mapping was carried out to detect and localize specific elements. The characteristic peaks of copper (Cu) and indium (In) were used to identify the CIPS ($CuInP_2S_6$) core. The presence of nitrogen (N) was attributed to the aptamer conjugation, while carbon (C) and oxygen (O) signals were considered from both the aptamer and organic linkers. Phosphorus (P) and sulfur (S) signals were analyzed to further verify the chemical composition of the CIPS component and crosslinking structure. Elemental distribution maps were generated and overlaid on the TEM images to visualize the uniformity and co-localization of the respective elements within the nanosheet structure. Data acquisition and analysis were carried out using accompanying software (e.g., Oxford Instruments AZtec or Bruker ESPRIT).

Cytotoxicity test. The cytotoxicities of the CIPS, C-TA, C-TA_E, and C-TA_H were evaluated using the CCK-8 assay. 1×10^4 cells were seeded in a 96-well plate and cultured at 37 °C in a humidified atmosphere with 5% CO_2. When the cell density reached 70–80%, the medium was replaced with a medium with a series of concentrations of the above-mentioned nanomaterials.

After 24 h or 48h incubation, the medium was replaced again with a solution containing 10% CCK-8 reagent and incubated for 2–3 h in a 37 °C incubator. Then, the absorbance of the solution was read at 450 nm using a Multiplate Reader.

dsDNA binding assay. The dsDNA concentration was measured using the Quant-iT PicoGreen Kit according to the manufacturer's protocol. Nanosheets with a series of concentrations were added to the test solution containing dsDNA (500 ng/mL), and then the Pico-green reagent was added. The dsDNA binding efficacy was calculated using the fluorescence intensity variation (Ex=480 nm; Em=520 nm).

NETs binding tests. Neutrophils were seeded into 24-well plates, with a covered glass at the bottom of the wells. To assess the binding effect of the nanomaterials on LPS-induced NET formation, neutrophils were stimulated with LPS and co-cultured with 2 μg/mL of the CIPS, C-TA, C-TA_E, and C-TA_H for 24 h. To evaluate the NET binding efficacy of the nanosheets, 2 μg/mL of CIPS, C-TA, C-TA_E, and C-TA_H was added with LPS stimulation and co-cultured for an additional 24 h. Immunofluorescence staining was performed using DAPI and CitH3 and then observed by a confocal laser scanning microscope (CLSM). Additionally, STYOX Green staining was applied and observed under a fluorescence microscope.

Antibacterial activity in vitro. (1) Contact antibacterial experiments: 100 μL CIPS, C-TA, C-TA_E, and C-TA_H solutions with a series of concentrations were put into a 2 mL Eppendorf tube. Then, E. coli or S. aureus suspension (1 mL) was added and incubated at 37 °C, shaking (220

rpm). Bacterial culture medium was collected at 10 h, and the OD value at 600 nm was measured. The bacterial growth curve was then plotted. (2) Surface antibacterial activity: Autoclaved nutrient agar (10 μL) was added to 60-mm Petri dishes to form a solid culture medium. Bacterial solutions of E. coli and S. aureus (200 μL) together with CIPS, C-TA, C-TA_E, and C-TA_H solutions (20 μL) with different concentrations were uniformly dispersed on the medium surface. After incubation at 37 °C for 24 h, images of the plates were recorded. Sterile and bacteria-contaminated media without nanosheets were used as negative controls, respectively.

Comparison of therapeutic effects of DNase I and C-TA_H: Rats were randomly assigned to four groups: Sham, OME + PBS, OME + DNase I, and OME + C-TA_H. DNase I was administered at a dose of 20 IU in 20 μL PBS per ear, and C-TA_H at 0.5 μg in 20 μL PBS per ear. The OME model was established as previously described. Following model induction, intratympanic injections were performed under microscopic guidance via the posterior-inferior or anterior-inferior quadrant of the tympanic membrane. Treatment groups received either free DNase I or C-TA_H, while the PBS group received vehicle alone. The Sham group received no injection. Auditory brainstem response (ABR) testing and micro-computed tomography (micro-CT) imaging were performed on days 2 and 9 post-injection to assess auditory function and structural changes in the middle ear, respectively.

ROS reduction studies. Raw264.7 cells were seeded into 96-well black plates (5×10^3 cells/well) and stimulated with LPS (1 μg/mL) for 24 h. Subsequently, CIPS, C-TA, C-TA_E, and C-TA_H

(2 μg/mL) were added to the cells and incubated for another 3 h. Finally, DCFH-DA was added, and the cells were incubated for 30 min. The fluorescent intensity of each well was quantified by a Multiwell Plate Reader (Ex=495 nm; Em=525 nm), and fluorescent images were recorded by CLSM (Ex=488 nm; Em=520 nm).

Flow characterization of immune cell populations. To characterize immune cell populations in the middle ear, lavage fluid was collected under sterile conditions and centrifuged at 800 rpm for 5 minutes at 4 °C to pellet the cells. The supernatant was discarded, and the cell pellet was resuspended in 100 μL of flow cytometry staining buffer (PBS containing 1% fetal bovine serum). Cells were incubated with the following antibodies for 30 minutes at 4 °C in the dark: anti-CD3 (T lymphocytes), anti-CD4 (helper T cells), anti-CD45RA (naive T cells), and anti-CD161 (NK T cells). After staining, cells were washed twice with staining buffer and resuspended in 200 μL of buffer for acquisition. Flow cytometric analysis was performed using a BD FACSCanto II cytometer (BD Biosciences). A minimum of 10,000 events were collected per sample. Data were analyzed using FlowJo v10 software (TreeStar).

Ex vivo fluorescence imaging. Rats in the OME groups received intratympanic injections of Cy5-labeled CIPS, C-TA, or C-TA$_H$ at a dose of 0.5 μg in 20 μL PBS per ear. A separate group injected with PBS alone served as the negative control. Injections were administered using a microsyringe under a surgical microscope, targeting the posterior-inferior or anterior-inferior quadrant of the tympanic membrane. The heads and major organs—including the heart, lungs,

liver, spleen, kidneys, and brain—were harvested at 1-, 3-, 7-, and 12-days post-injection and subjected to *ex vivo* fluorescence imaging to assess biodistribution.

2. Analysis of the MEE from patients with OME

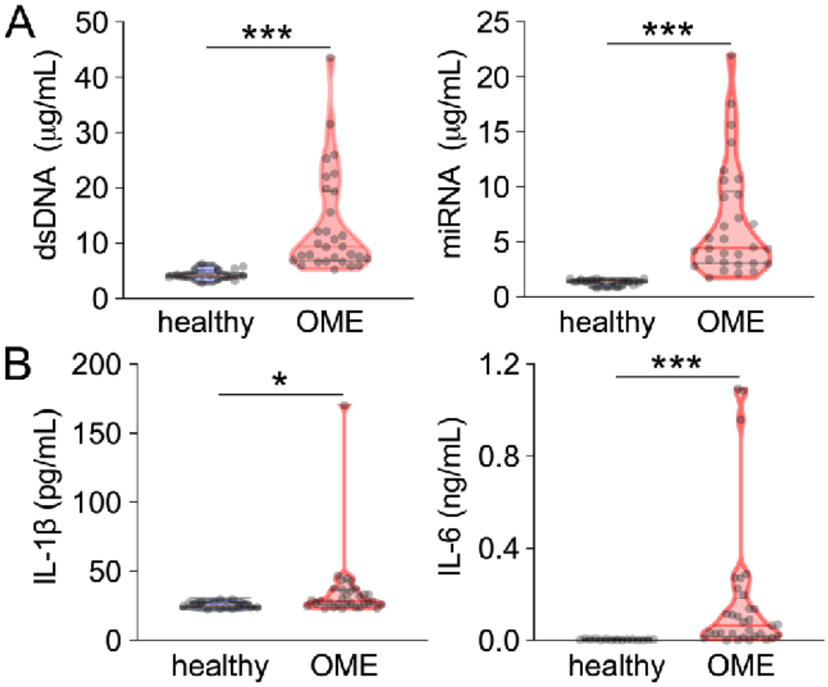

Figure S1. The levels of dsDNA, miRNA, IL-6, and IL-1β in MEE of patients with otitis media with effusion (OME) (n=31) and plasma of healthy volunteers (n=10). Data are presented as means ± SD, and assessed by the student-t test of variance (*p<0.05, **p<0.01, ***p<0.001).

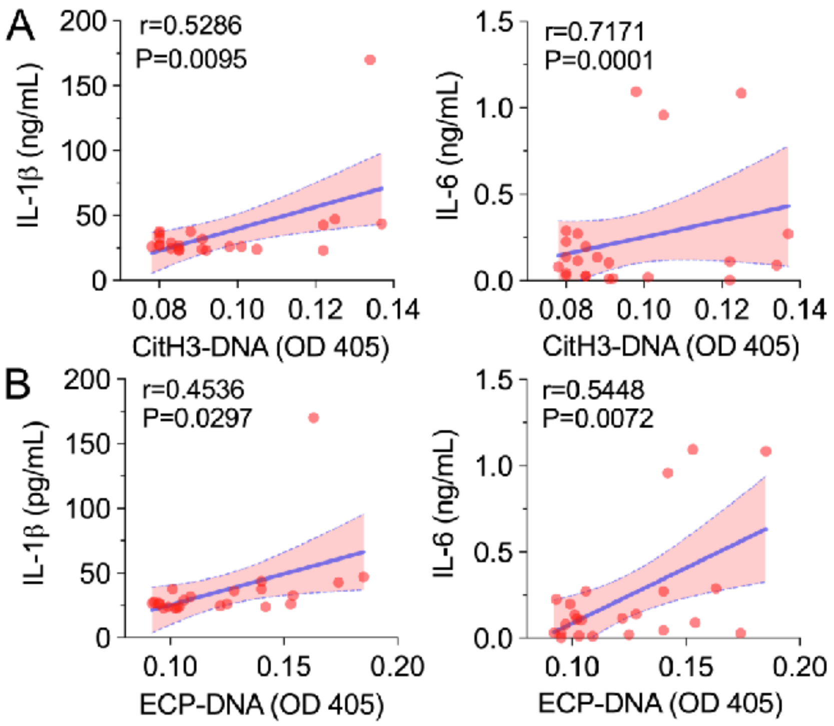

Figure S2. The relationship between NETs and EETs and the cytokine levels of patients with OME. (A) The correlation between the CitH3-DNA and IL-1β, IL-6 levels in the exudation from the middle ear (MEE) of OME patients. (B) The correlation between the ECP-DNA and IL-1β, IL-6 levels in the MEE of OME patients. The correlation of clinical data was assessed by the Spearman correlation coefficient (r).

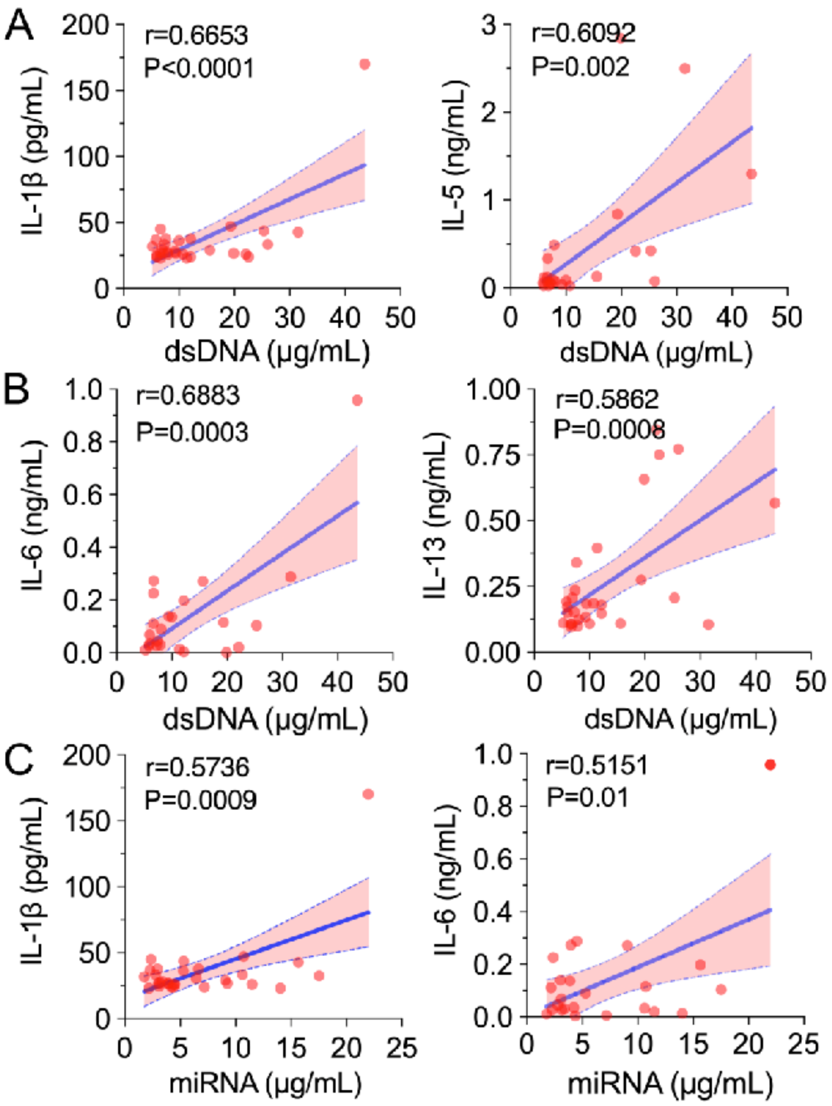

Figure S3. The relationship between dsDNA, miRNA, and cytokine levels in MEE of patients with OME. (A, B) The correlation between the dsDNA and IL-5, IL-13, IL-6, and IL-1β levels in the MEE of patients with OME. (C) The correlation between the miRNA and IL-6, IL-1β levels in the MEE of patients with OME. The correlation of clinical data was assessed by the Spearman correlation coefficient (r).

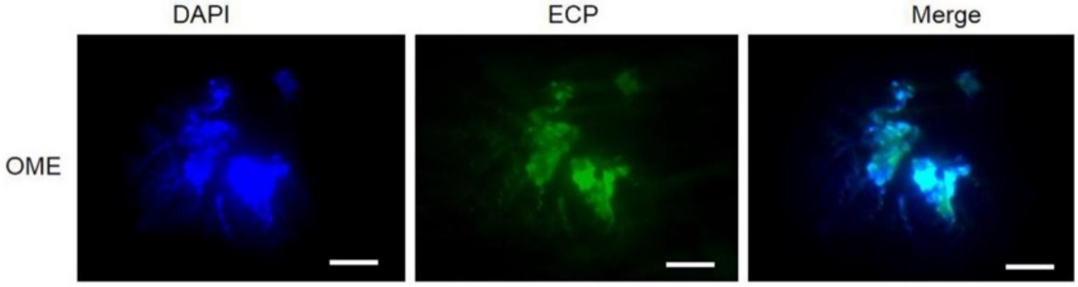

Figure S4. Representative immunofluorescence images of DAPI and ECP staining of MEE smears from OME patients. ECP, Eosinophil cationic protein. Scale bars: 200 μm.

3. Synthesis of functionalized nanosheets

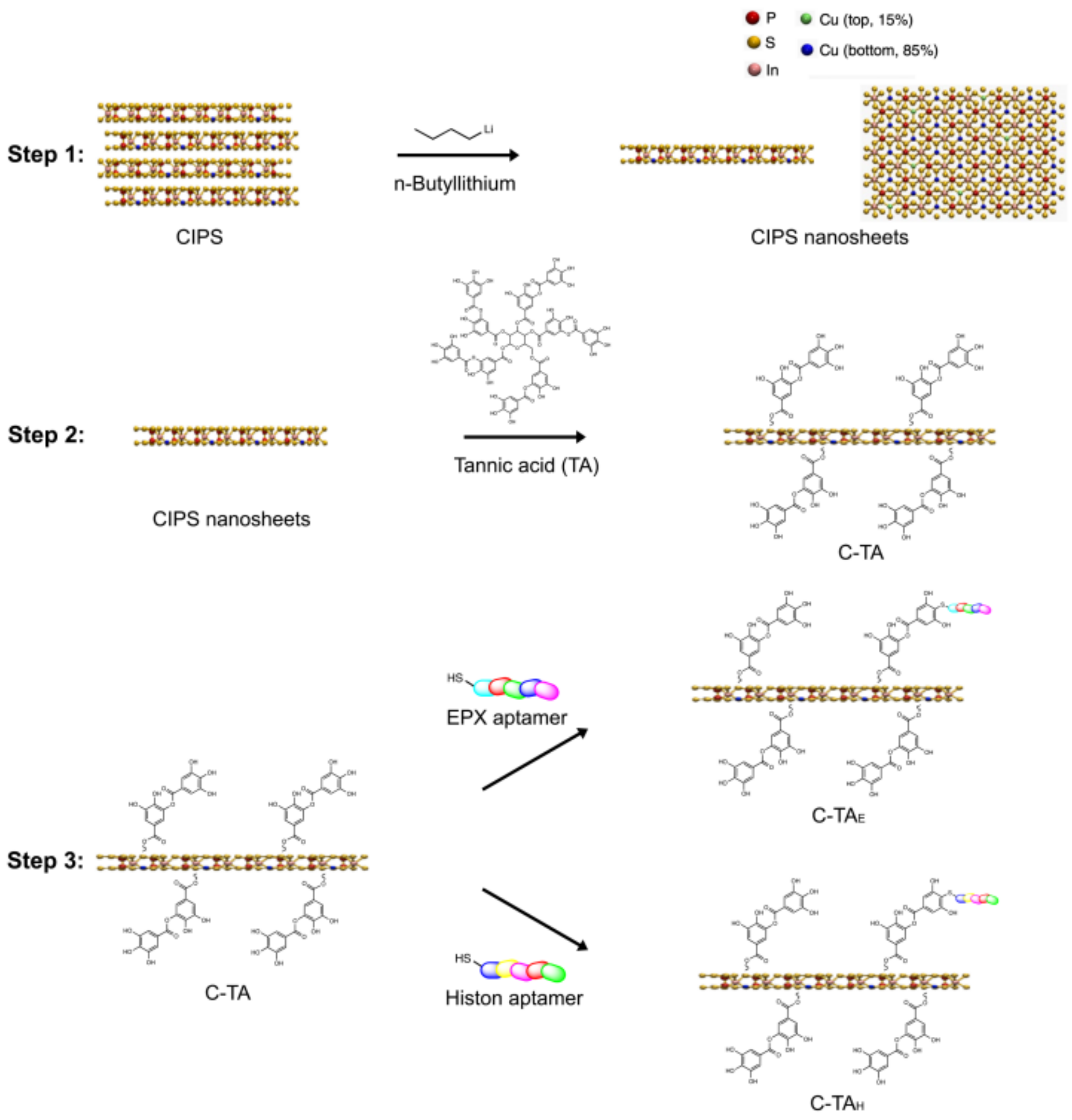

Figure S5. Synthesis routes for CIPS, C-TA, C-TA_E, and C-TA_H. Detailed methods are provided in the Experimental section above.

4. NMR of functionalized nanosheets

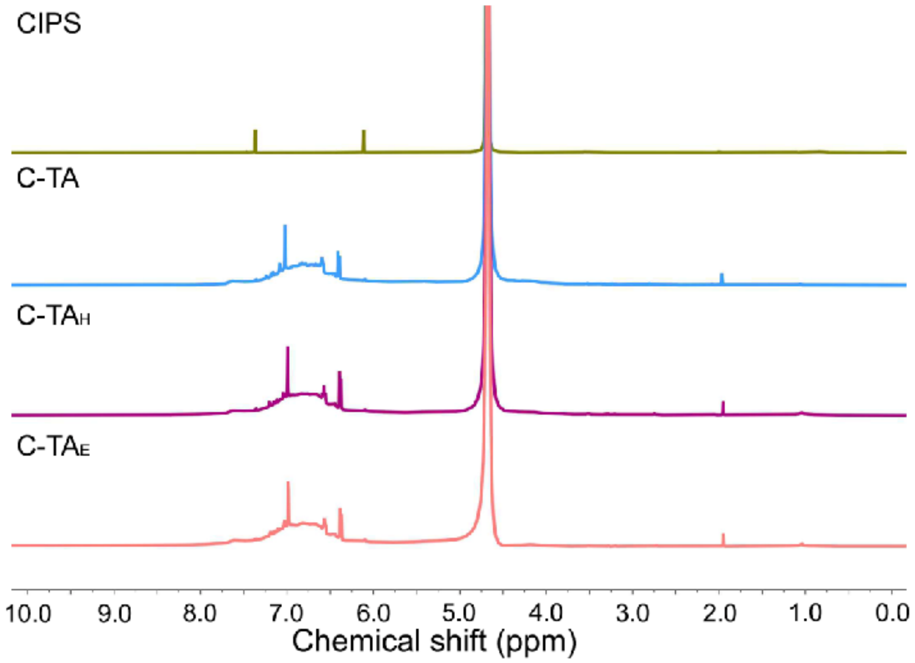

Figure S6. ^{1}H NMR data of CIPS, C-TA, C-TA$_H$, and C-TA$_E$ in D_2O. Signals at 4.7 ppm are attributed to the protons of D_2O. Signals between 6.2 and 7.2 are corresponded to the protons of tannic acids (TA).

5. FTIR of functionalized nanosheets

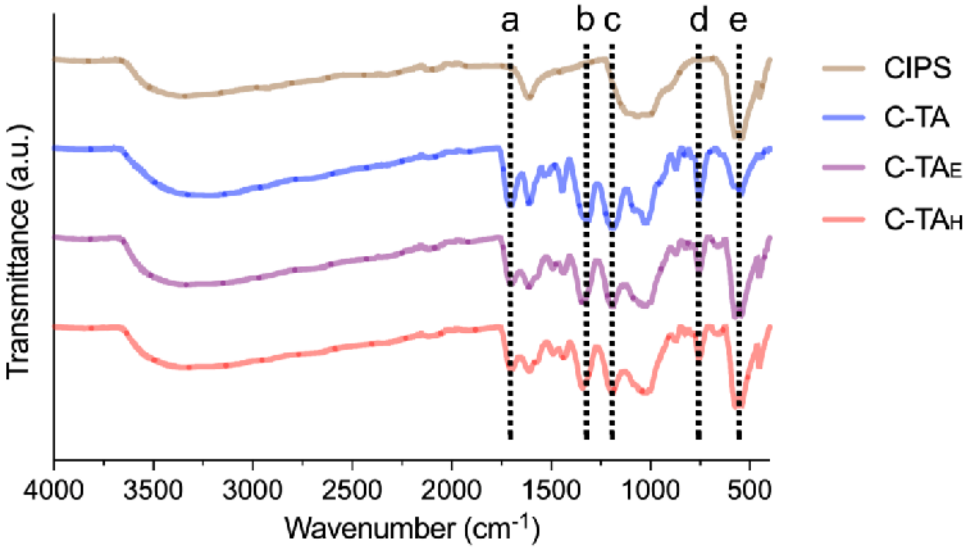

Figure S7. FTIR results of CIPS, C-TA, C-TA_E, and C-TA_H.

The IR spectra confirmed the successful modification of TA on CIPS nanosheets. Characteristic absorbance peaks a (1700 cm^{-1}), b (1310 cm^{-1}), c (1180 cm^{-1}), d (750 cm^{-1}), and e (550 cm^{-1}) corresponded to the C=O (a), Ph-O (b, c), Ph-H (d), and P-S (e) bonds of TA.

6. UV-vis absorption

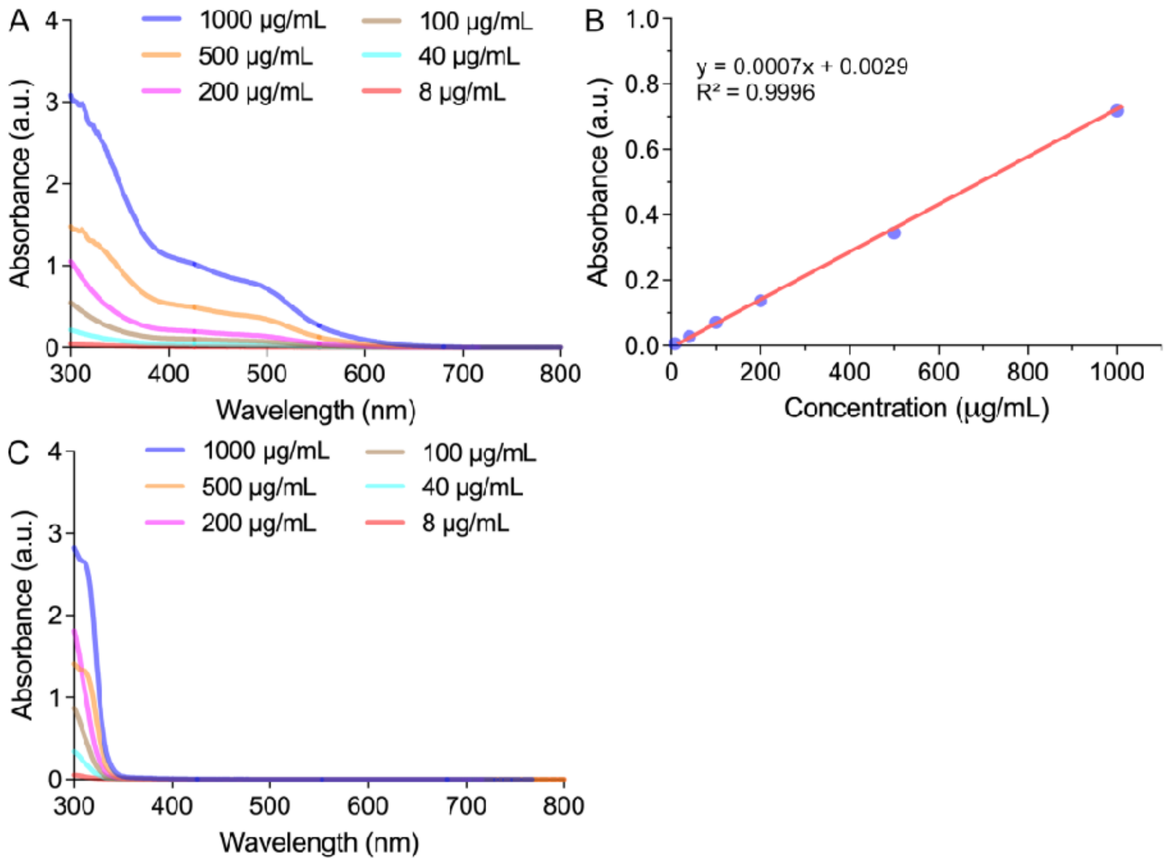

Figure S8. (A) UV-vis absorption of CIPS with a series of concentrations (8 μg/mL–1000 μg/mL). (B) Standard curve of CIPS with absorbance at 500 nm. (C) UV-vis absorption of TA with a series of concentrations (8 μg/mL–1000 μg/mL).

7. Determination of aptamer content

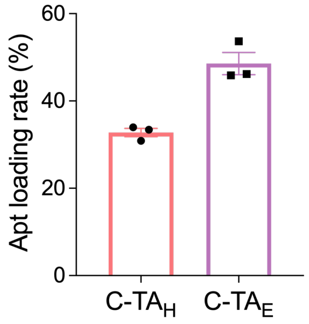

Figure S9. The amount of aptamer conjugated to the nanosheets C-TAE and C-TAH.。 Data are presented as means ± SD, and assessed by the student-t test of variance.

8. Elemental Mapping Data

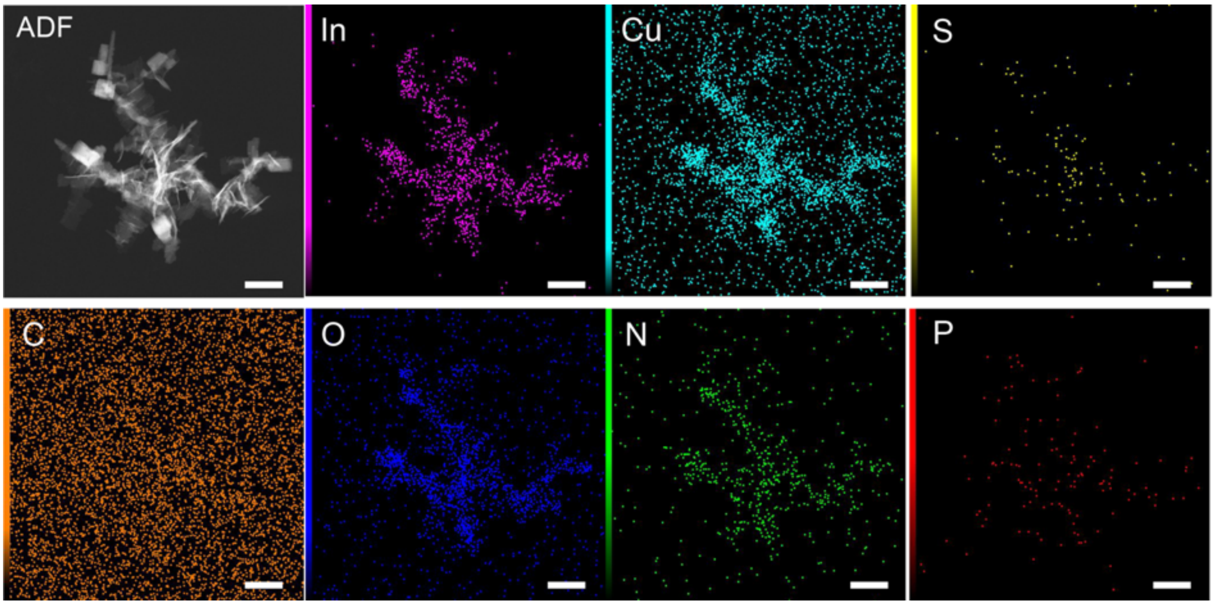

Figure S10. Elemental Mapping Data of C-TA$_H$ by Energy Dispersive Spectroscopy (EDS). Scale bar, 100 nm.

7. DLS and zeta potential

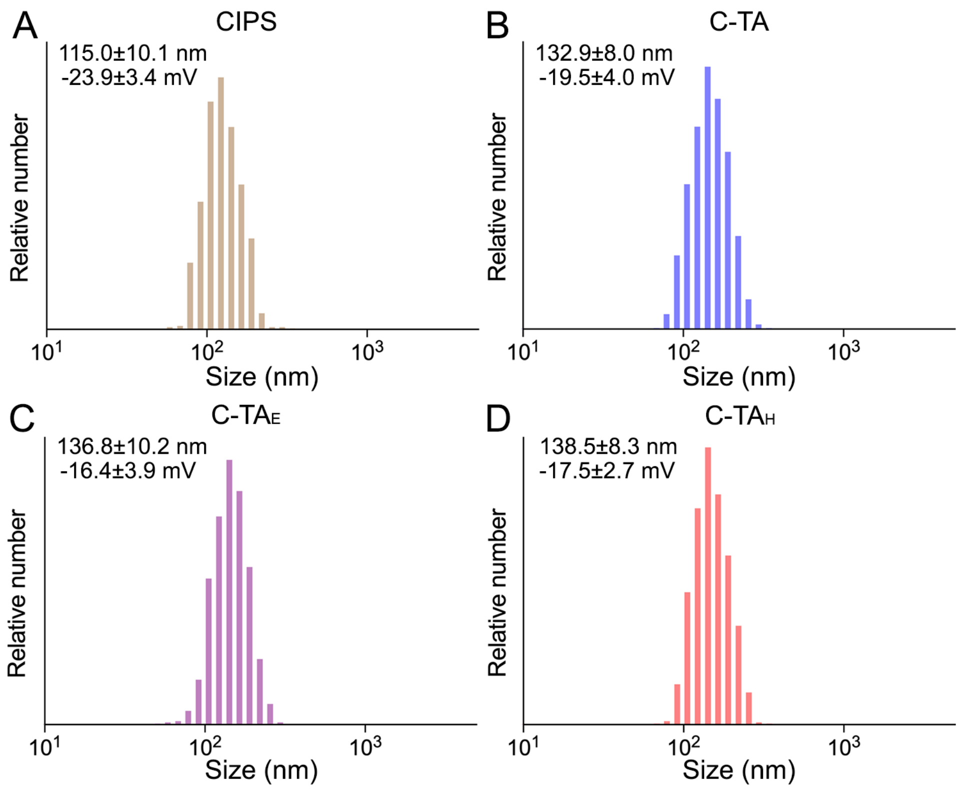

Figure S11. DLS and zeta potential of CIPS, C-TA, C-TA$_E$, and C-TA$_H$ in PBS (7.4).

8. Cytotoxicity

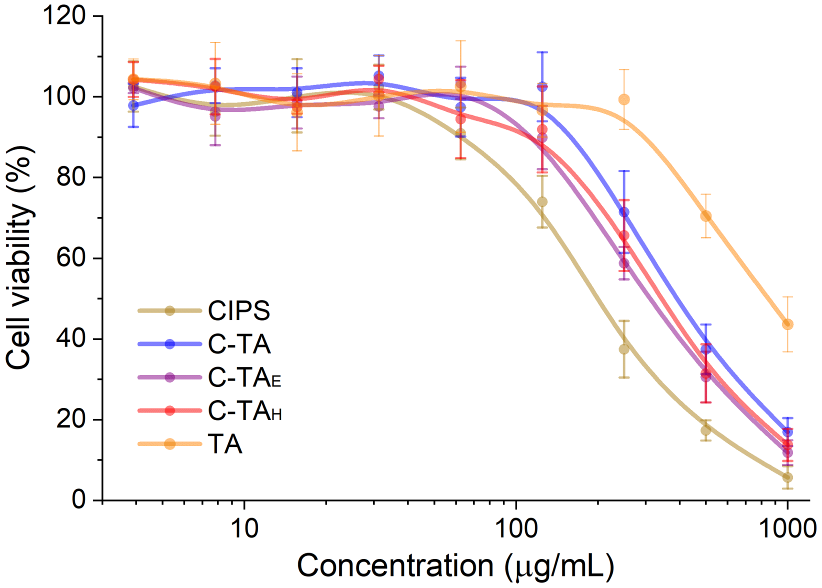

Figure S12. Cell viability of different groups after 48 h treatment with CIPS, C-TA, C-TA_E, C-TA_H, and TA at a series of concentrations. Cells without any treatment were used as a negative control. Data represent mean ± S.D. (n=3).

9. EET binding study

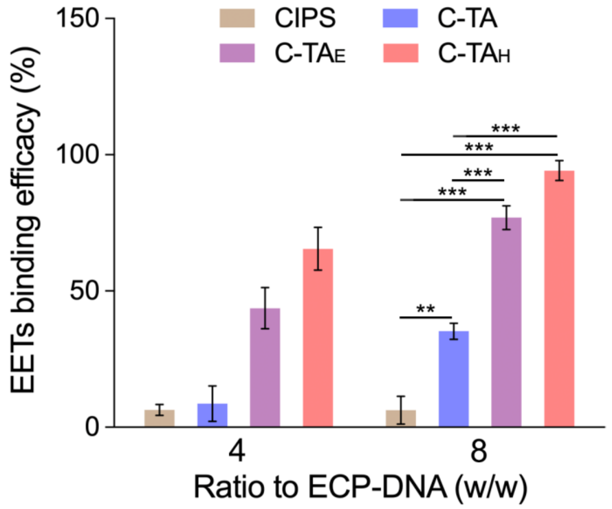

Figure S13. Quantitative EETs-binding capacities of CIPS, C-TA, C-TA$_E$, and C-TA$_H$. Data are presented as means ± SD, and assessed by one-way ANOVA with Tukey's multiple comparison test. (One-way ANOVA, *p<0.05*,* **p<0.01 *and* ***p<0.001)

10. q-PCR tests

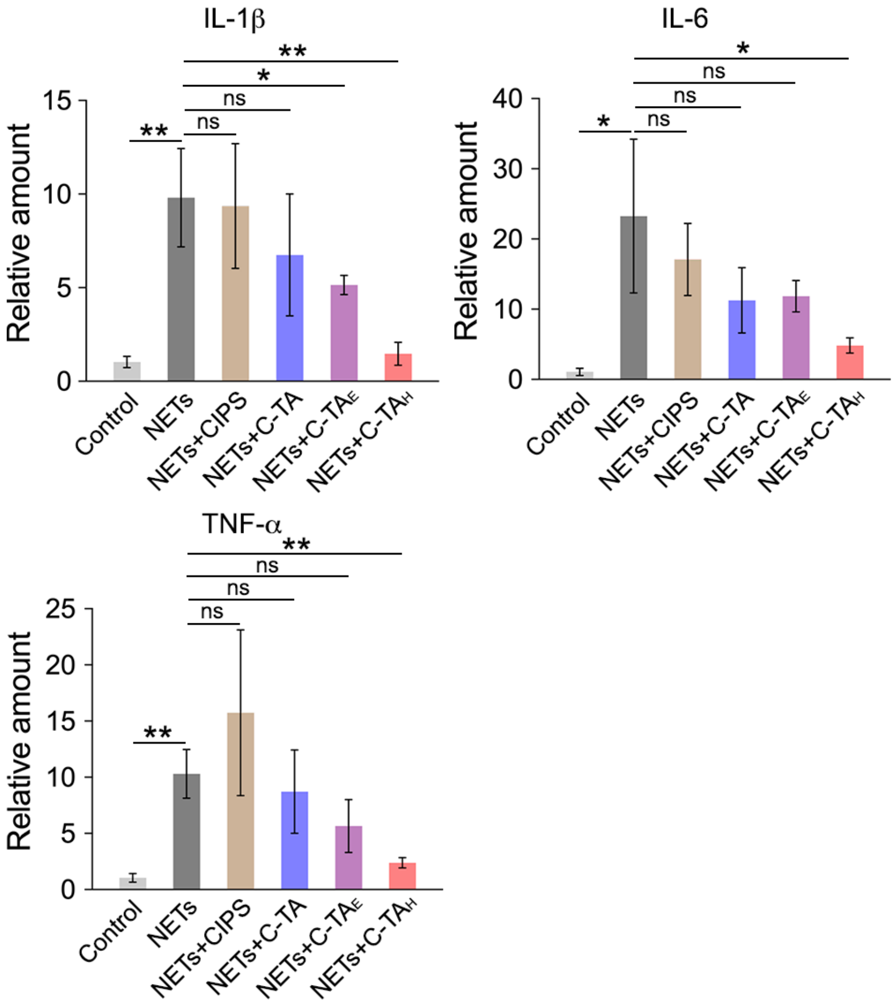

Figure S14. The relative mRNA expression levels of IL-1β, IL-6, and TNF-α in the Raw264.7 cells treated with NETs, NETs+CIPS, NETs+C-TA, NETs+C-TA$_E$, and NETs+C-TA$_H$. Data are presented as means ± SD, and assessed by one-way ANOVA with Tukey's multiple comparison test. (ns represents not significant, *p<0.05*,* **p<0.01 *and* ***p<0.001)

11. Anti-bacterial efficacy

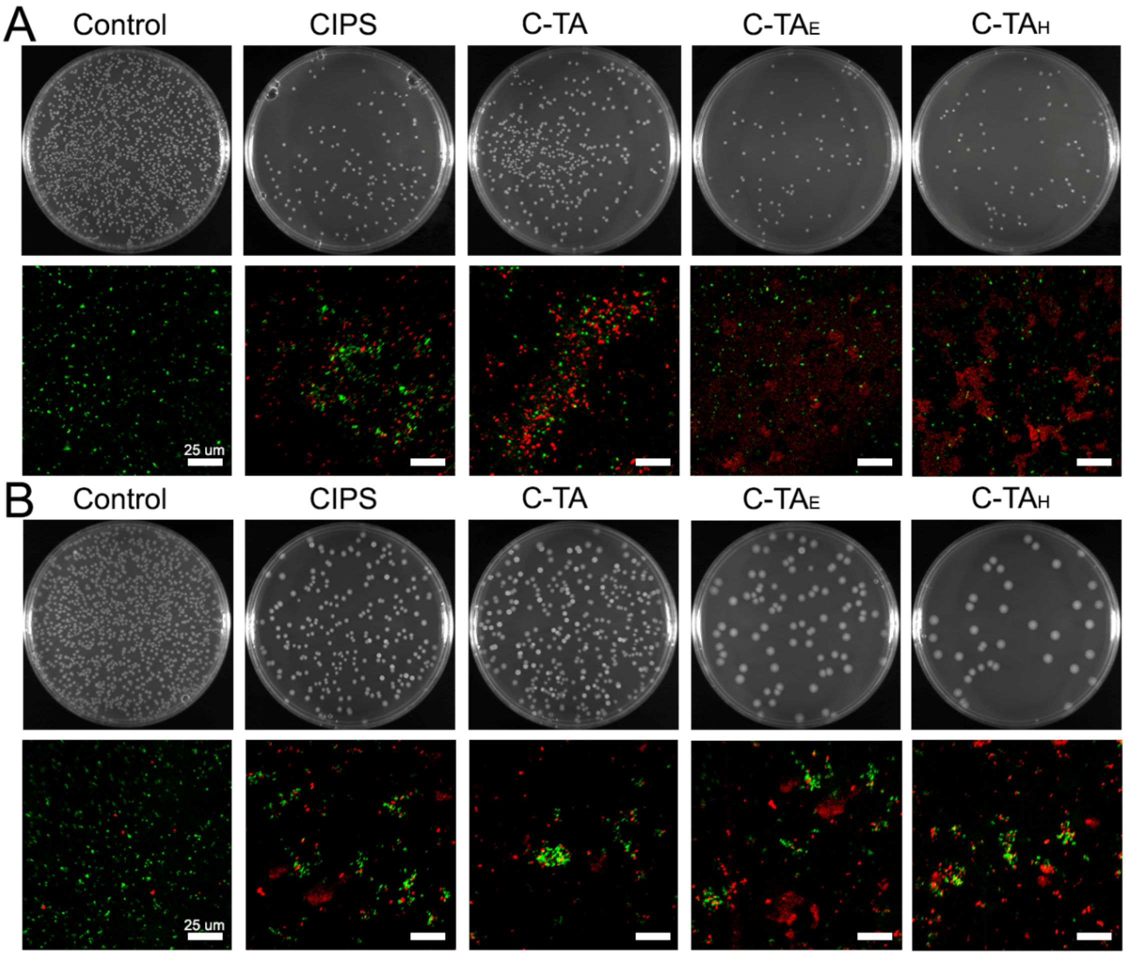

Figure S15. (A) Light microscopy images of E. coli and CLSM images of live/dead staining of bacteria (green: live; red: dead) after a 24-hour incubation with nanosheets. Scale bars: 100 nm. (B) Light microscopy images of S. aureus and CLSM images of live/dead staining of bacteria (green: live; red: dead) after 24-hour incubation with nanosheets. Scale bars: 25 μm.

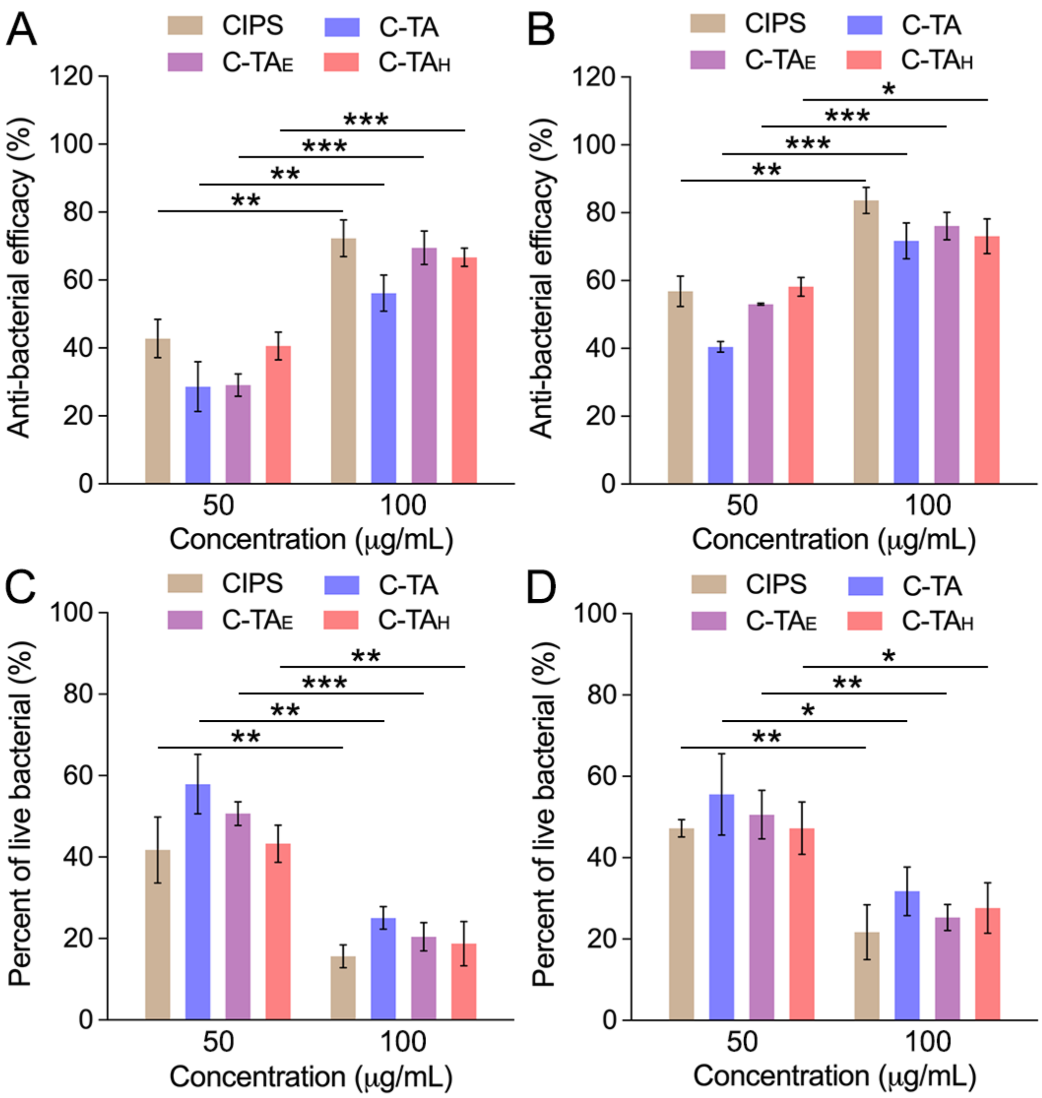

Figure S16. (A) Quantitative 24-hour antibacterial efficacy of E. coli based on the culture medium's OD620 value. (B) Quantitative 24-hour antibacterial efficacy of S. aureus based on the culture medium's OD620 value. (C) The quantitative ratio of live bacteria (E. coli) after 24-hour incubation based on CLSM images. (D) The quantitative ratio of live bacteria (S. aureus) after 24-hour incubation based on CLSM images. Data are presented as means ± SD, and assessed by one-way ANOVA with Tukey's multiple comparison test. (*p<0.05, **p<0.01 *and* ***p<0.001)

12. Antioxidant studies

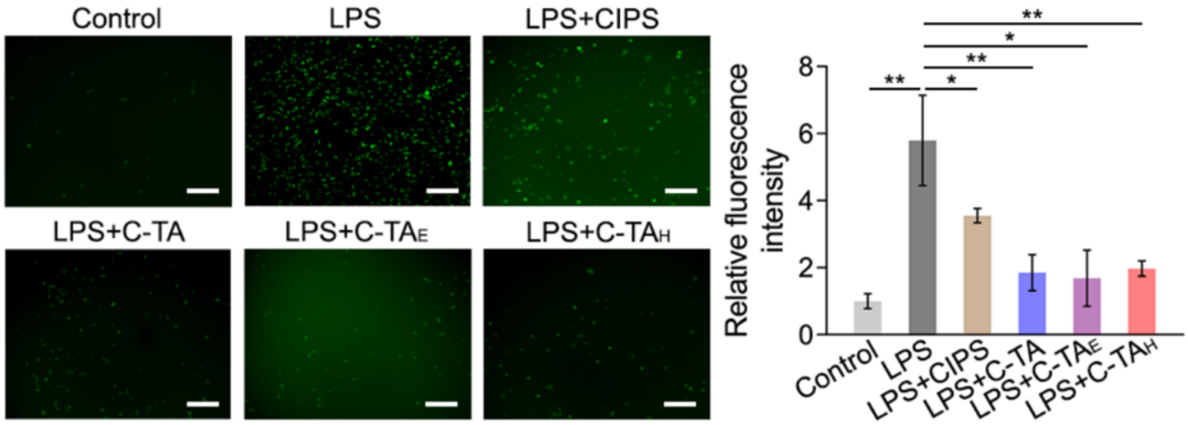

Figure S17. The CLSM images of DCFH fluorescence in LPS-treated cells incubated with CIPS, C-TA, C-TA$_E$, and C-TA$_H$ in PBS. Quantitative DCFH fluorescence intensities in CLSM images. Scale bars: 100 μm. Data are presented as means ± SD, and assessed by one-way ANOVA with Tukey's multiple comparison test. (**p*<0.05*,* **p<0.01 *and* ****p*<0.001)

13. Biodistribution studies

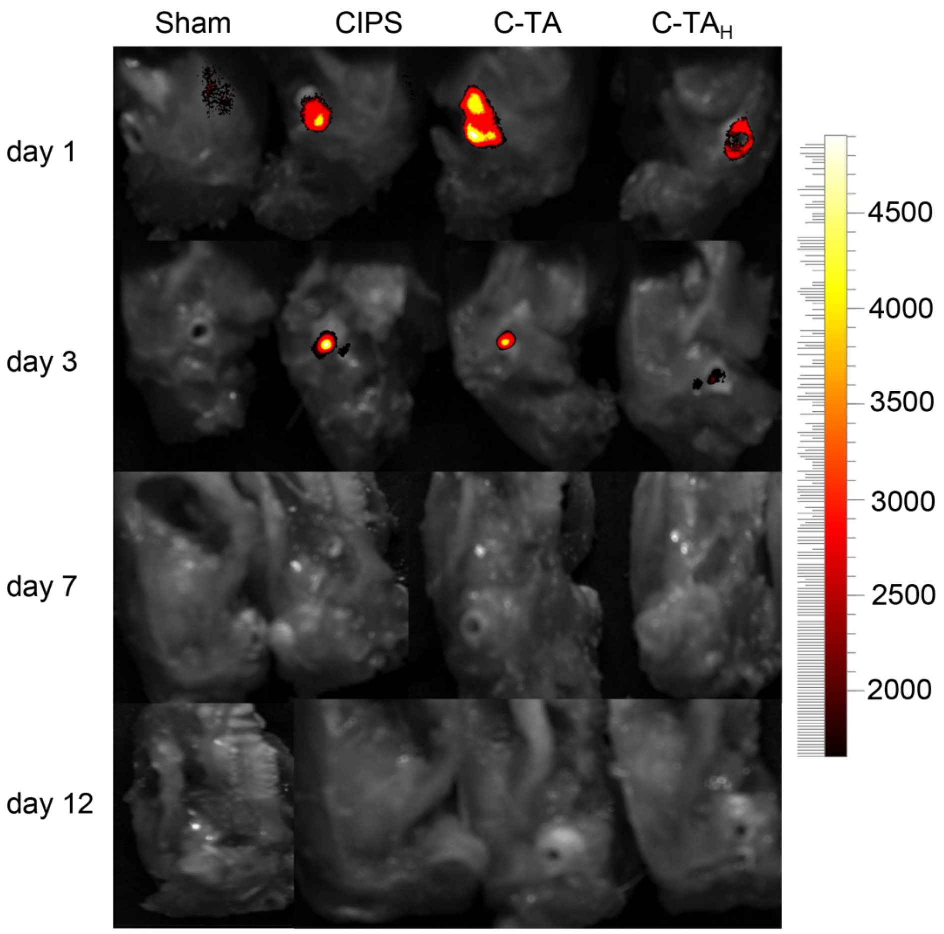

Figure S18. Biodistribution and biodegradation of CIPS, C-TA, and C-TA$_H$ in the middle ear of OME rats after local administration. CIPS, C-TA, and C-TA$_H$ were labeled by Cy5.

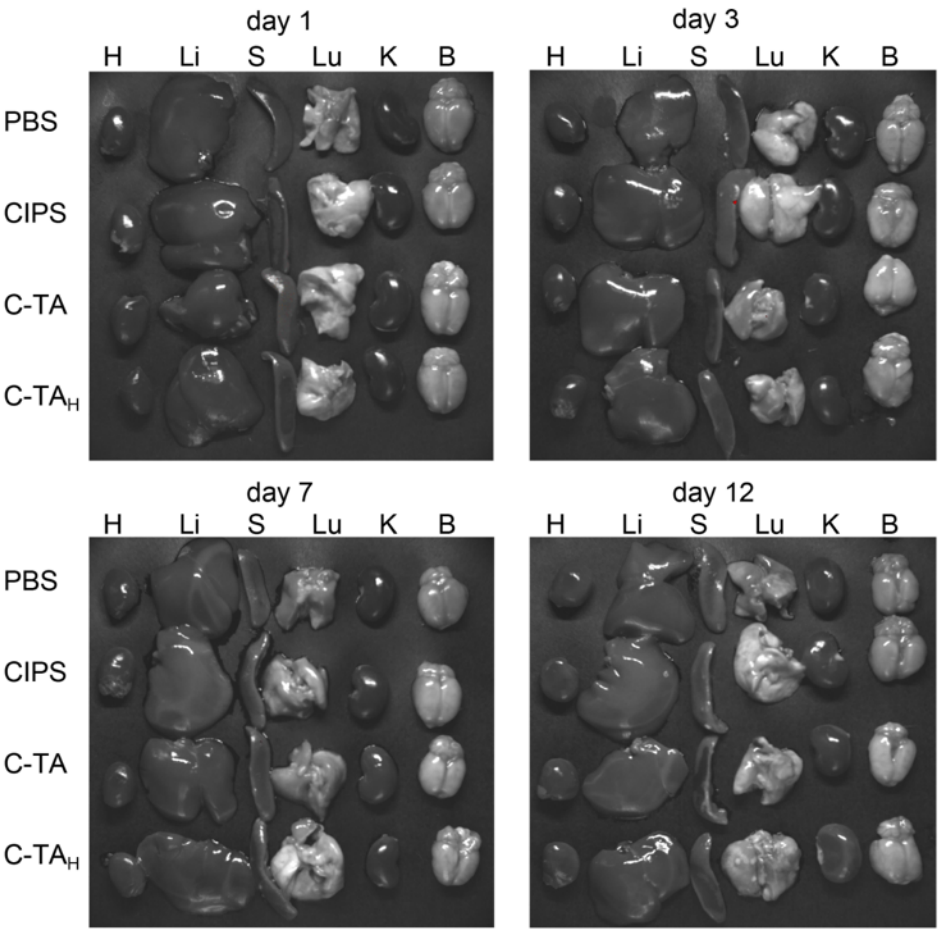

Figure S19. Biodistribution and biodegradation of CIPS, C-TA, and C-TA$_H$ in the organs of OME rats. (From left to right are the heart, liver, spleen, lung, kidney and brain). CIPS, C-TA, and C-TA$_H$ were labeled by Cy5.

14. Auditory brainstem response tests

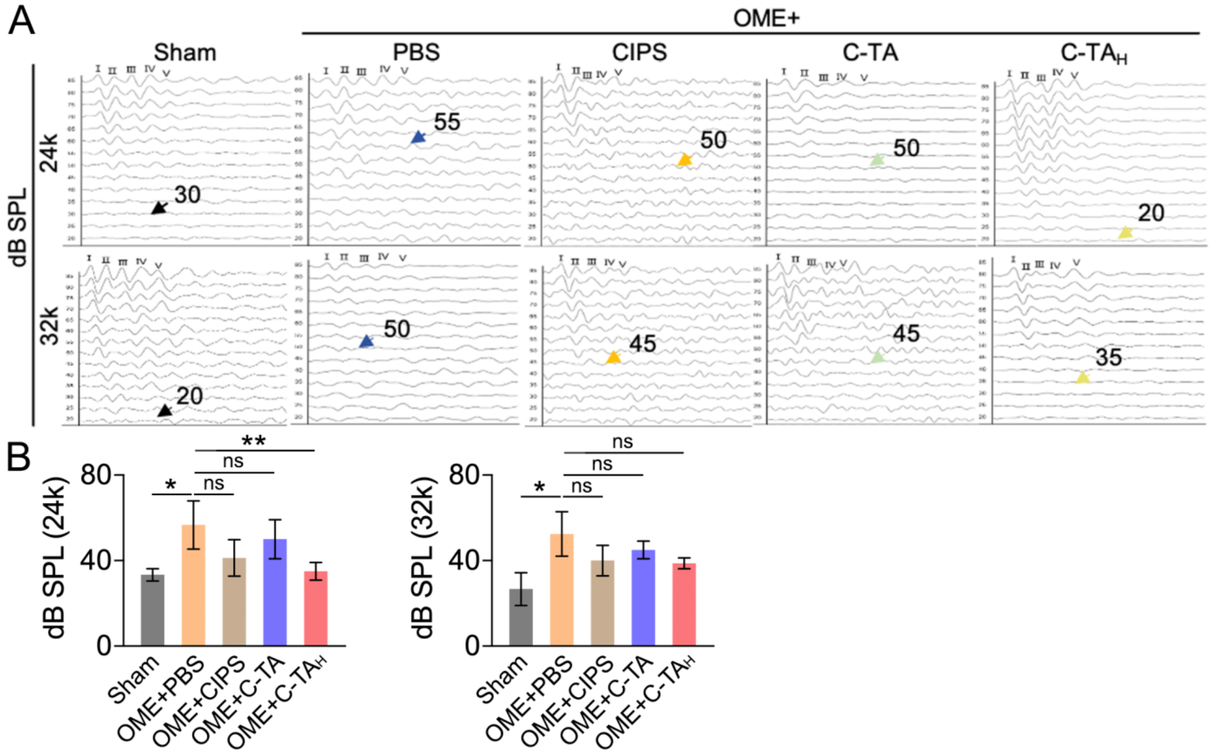

Figure S20. Auditory brainstem response (ABR) levels and threshold value of OME rats in different groups. Data are presented as means ± SD, and assessed by one-way ANOVA with Tukey's multiple comparison test. (ns represents not significant, **p*<0.05 and ***p*<0.01)

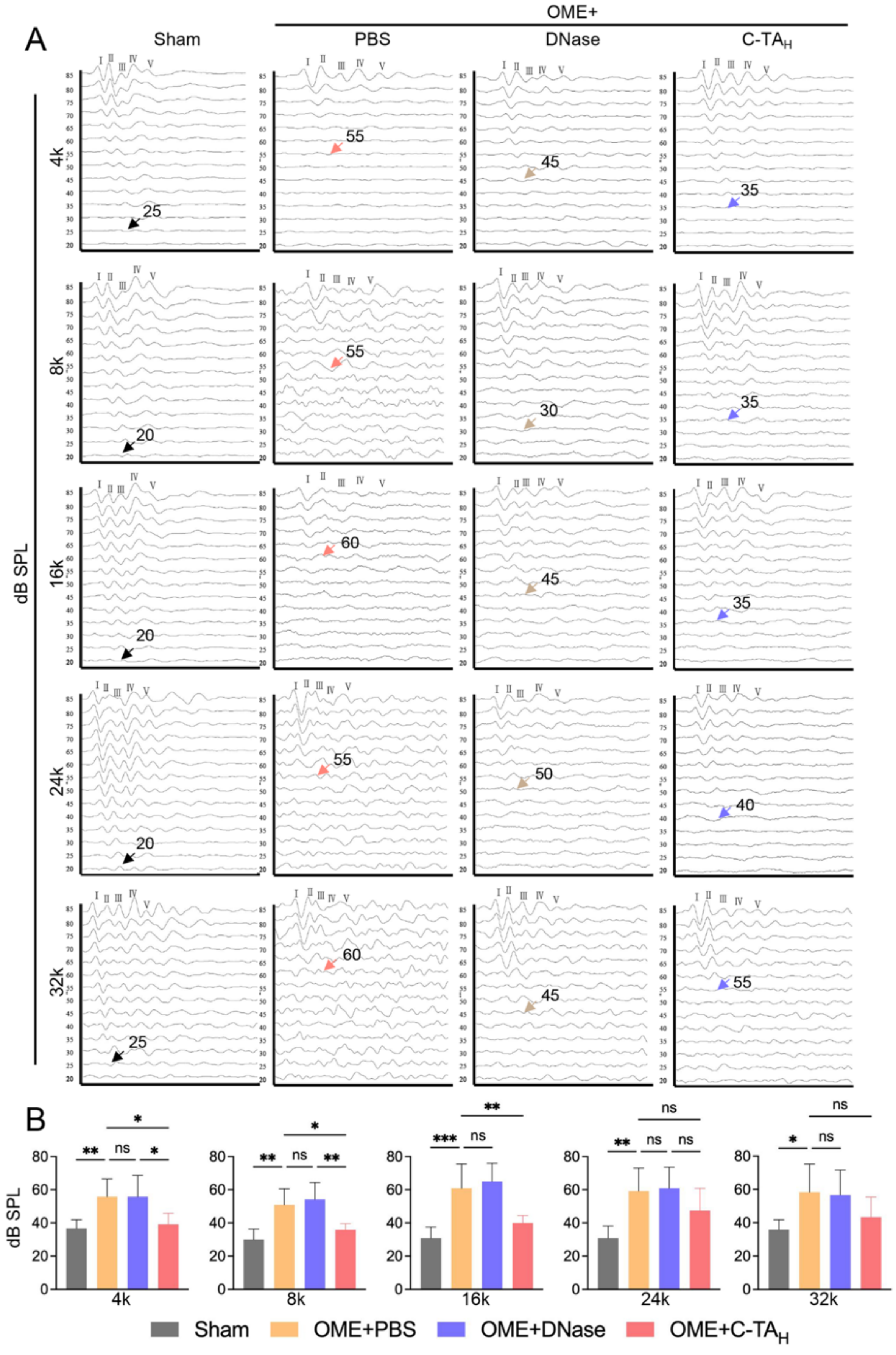
A
OME+
Sham
PBS
DNase
C-TA$_H$
dB SPL
4k
8k
16k
24k
32k
25
55
45
35
20
55
30
35
20
60
45
35
20
55
50
40
25
60
45
55
B
dB SPL
4k
8k
16k
24k
32k
ns
Sham
OME+PBS
OME+DNase
OME+C-TA$_H$

Figure S21. Auditory brainstem response (ABR) levels and threshold values in OME rats from different treatment groups on Day 2. Data are presented as means ± SD, and assessed by one-way ANOVA with Tukey's multiple comparison test. (ns represents not significant, *$p<0.05$, **$p<0.01$ and ***$p<0.001$)

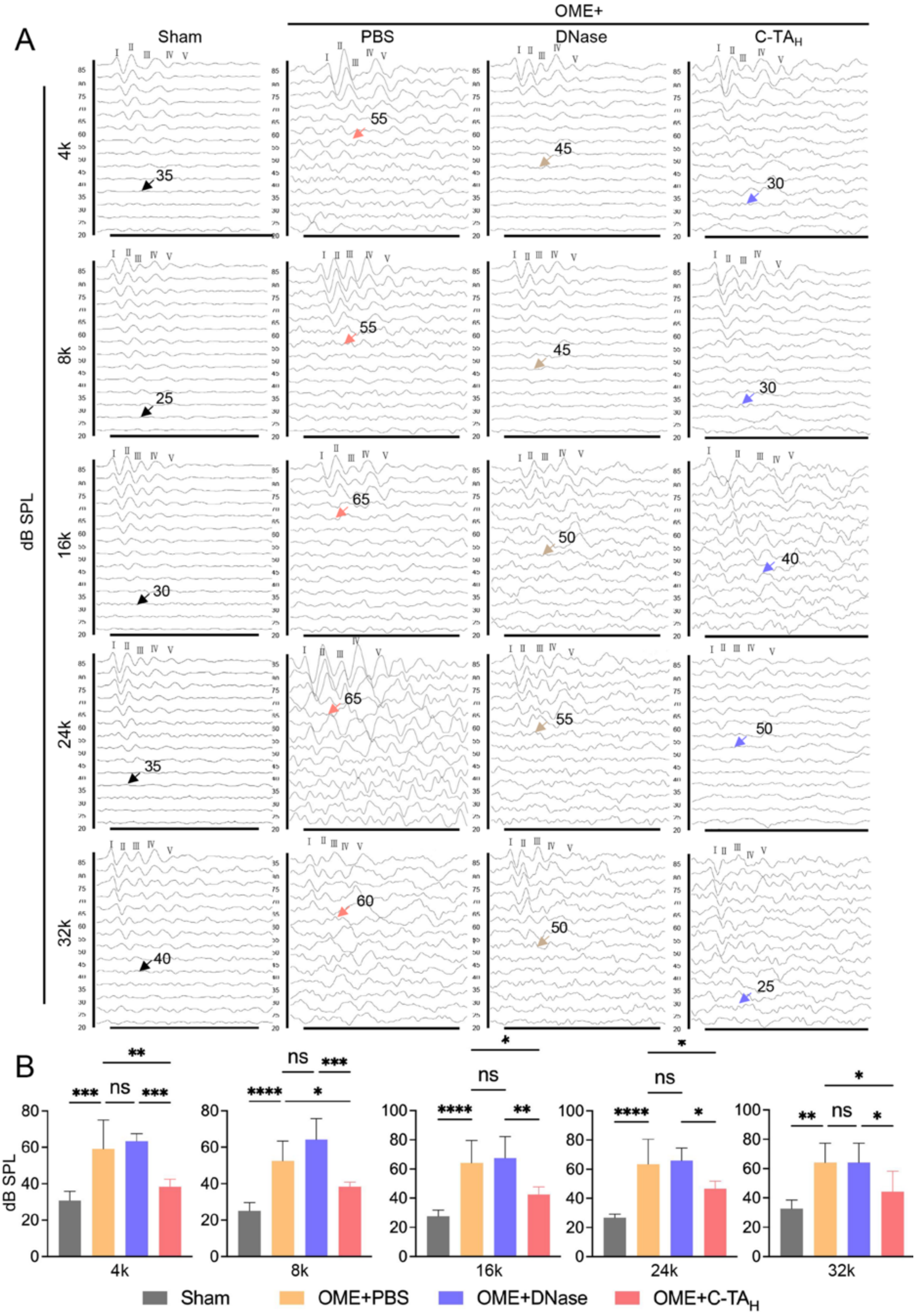
A
OME+
Sham
PBS
DNase
C-TAH
dB SPL
4k
8k
16k
24k
32k
35
55
45
30
25
55
45
30
30
65
50
40
35
65
55
50
40
60
50
25
B
dB SPL
4k
8k
16k
24k
32k
Sham
OME+PBS
OME+DNase
OME+C-TAH

Figure S22. Auditory Brainstem Response (ABR) Levels and threshold values in OME Rats from different treatment groups on day 9. Data are presented as means ± SD, and assessed by one-way ANOVA with Tukey's multiple comparison test. (ns represents not significant, $*p < 0.05$, $**p < 0.01$)

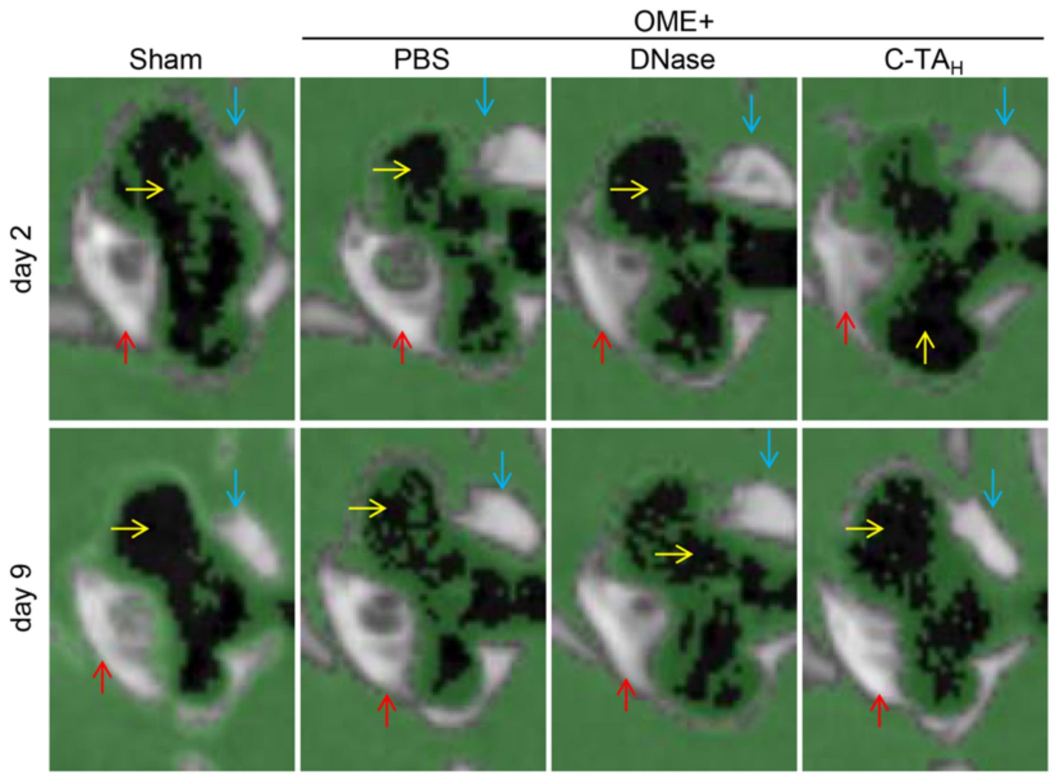

Figure S23. micro-CT images of ears of experimental rats. (The red arrow indicates the cochlea, the yellow indicates the middle ear cavity, and the blue indicates the mastoid).

15. Analysis of middle ear lavage fluid

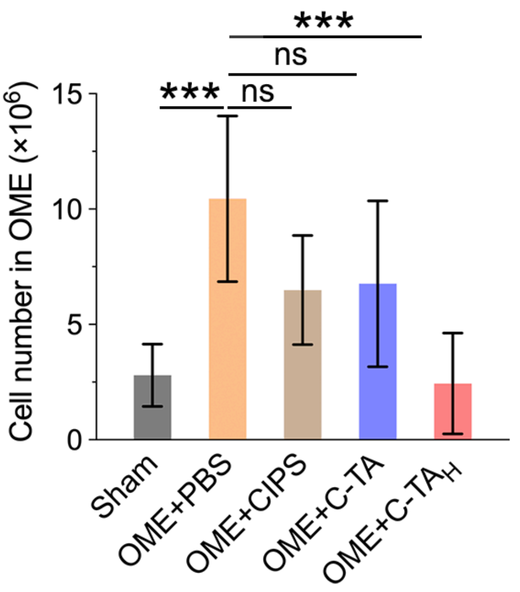

Figure S24. The number of total cells in the middle ear lavage fluid of rats from different treatment groups. Data are presented as means ± SD, and assessed by one-way ANOVA with Tukey's multiple comparison test. (ns represents not significant, **p*<0.05, **p<0.01 *and* ****p*<0.001)

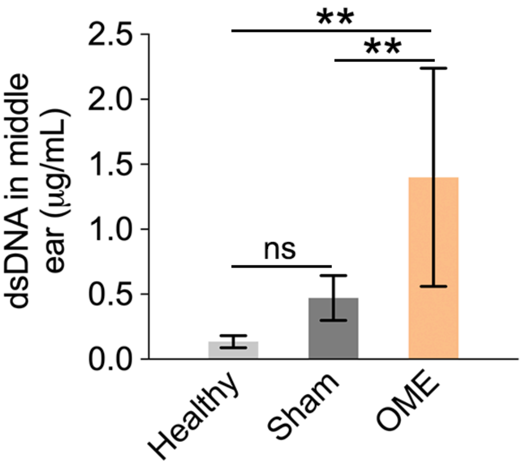

Figure S25. dsDNA concentration in middle ear lavage fluid of rats in different treatment groups. Data are presented as means ± SD; assessed by one-way ANOVA with Tukey's multiple comparison test. (ns represents not significant, **p*<0.05 and ***p*<0.01.)

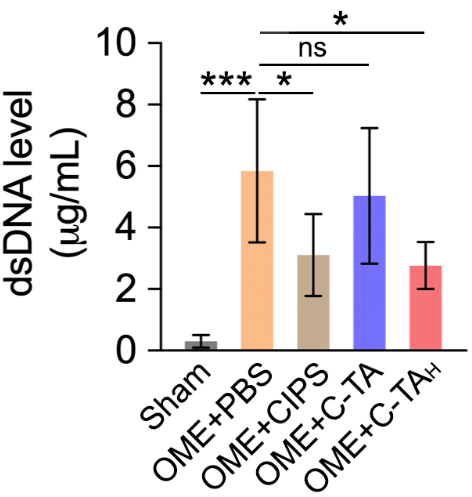

Figure S26. dsDNA concentration in middle ear lavage fluid of rats in different treatment groups. Data are presented as means ± SD, and assessed by one-way ANOVA with Tukey's multiple comparison test. (ns represents not significant, *p<0.05 and **p<0.01 and ***p<0.001)

16. Immunostaining slices of middle ear lavage fluid smears

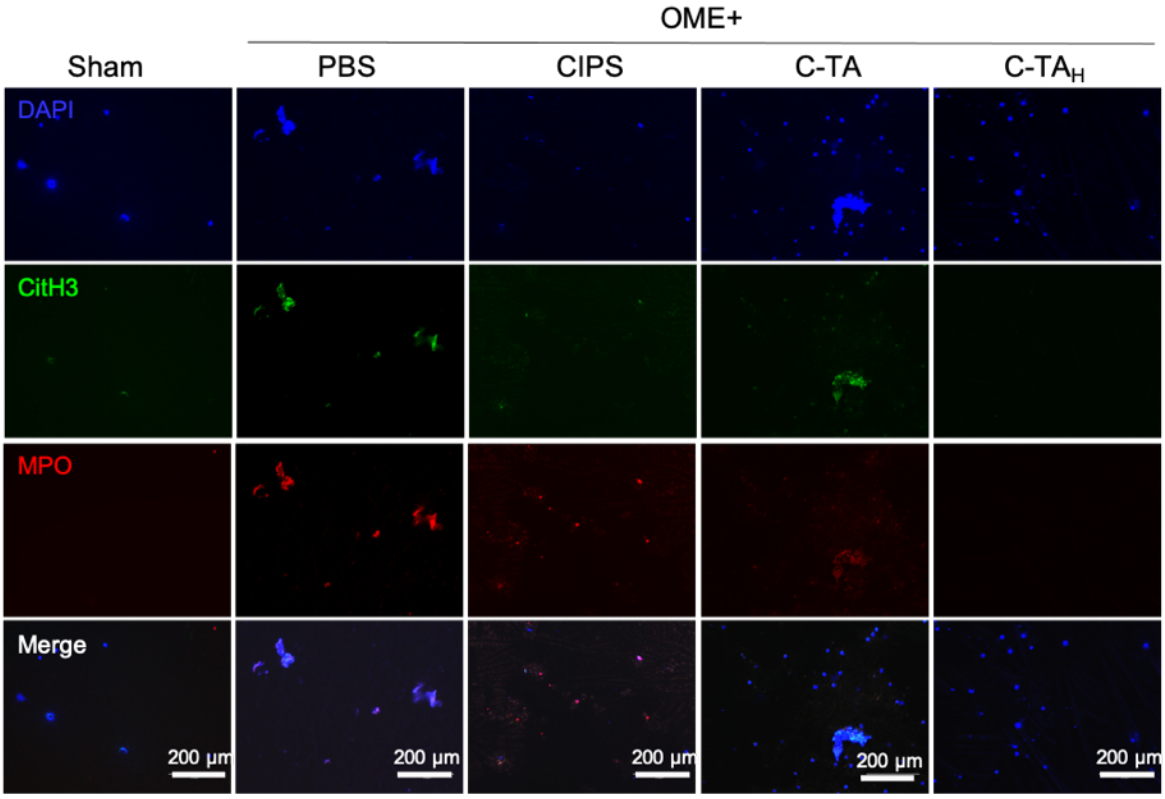

Figure S27. Representative immunofluorescence images of DAPI, CitH3, and MPO immunostaining of the middle ear lavage fluid smears of rats from different treatment groups.

17. Immunostaining staining of middle ear tissues

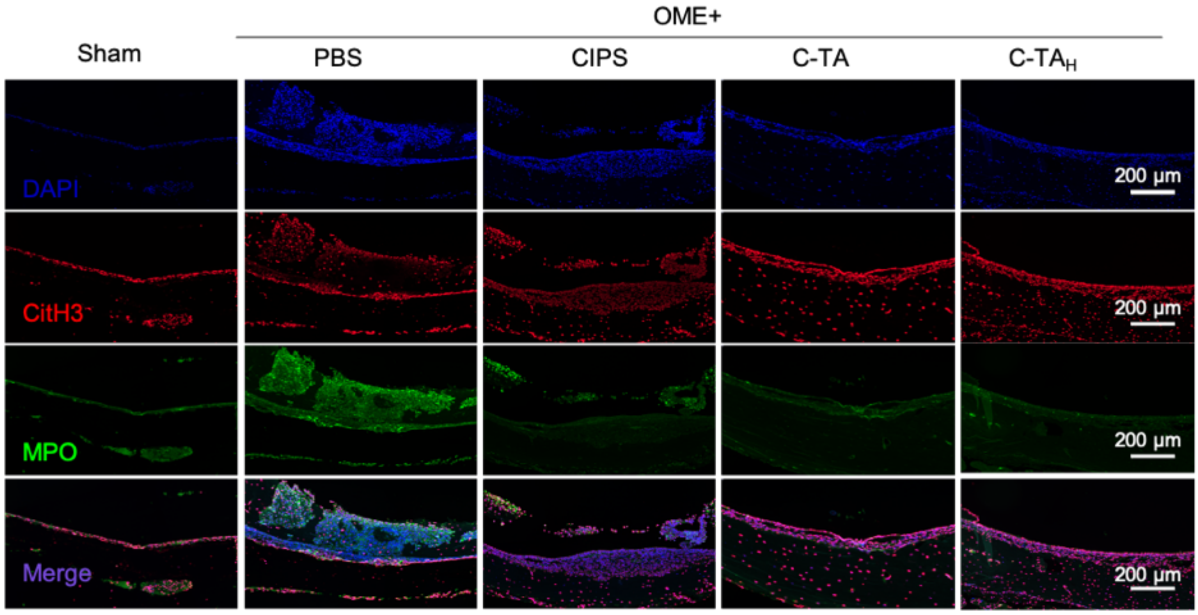

Figure S28. Representative immunofluorescence images of CitH3, ECP, and DAPI staining of middle ear tissues from OME rats in different treatment groups.

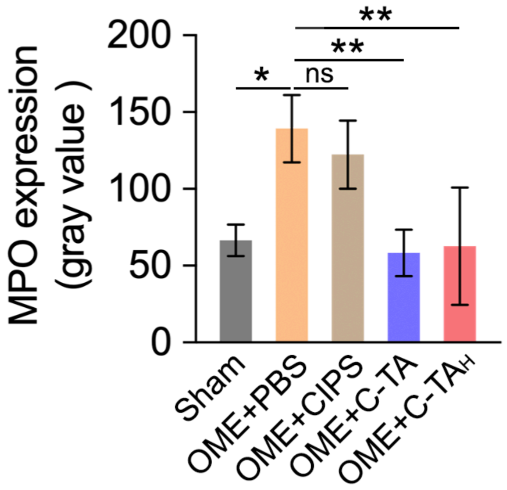

Figure S29. The relative mean fluorescence intensity of MPO was calculated using Image J software. Data are presented as means ± SD, and assessed by one-way ANOVA with Tukey's multiple comparison test. (ns represents not significant, *p<0.05 and **p<0.01 and ***p<0.001)

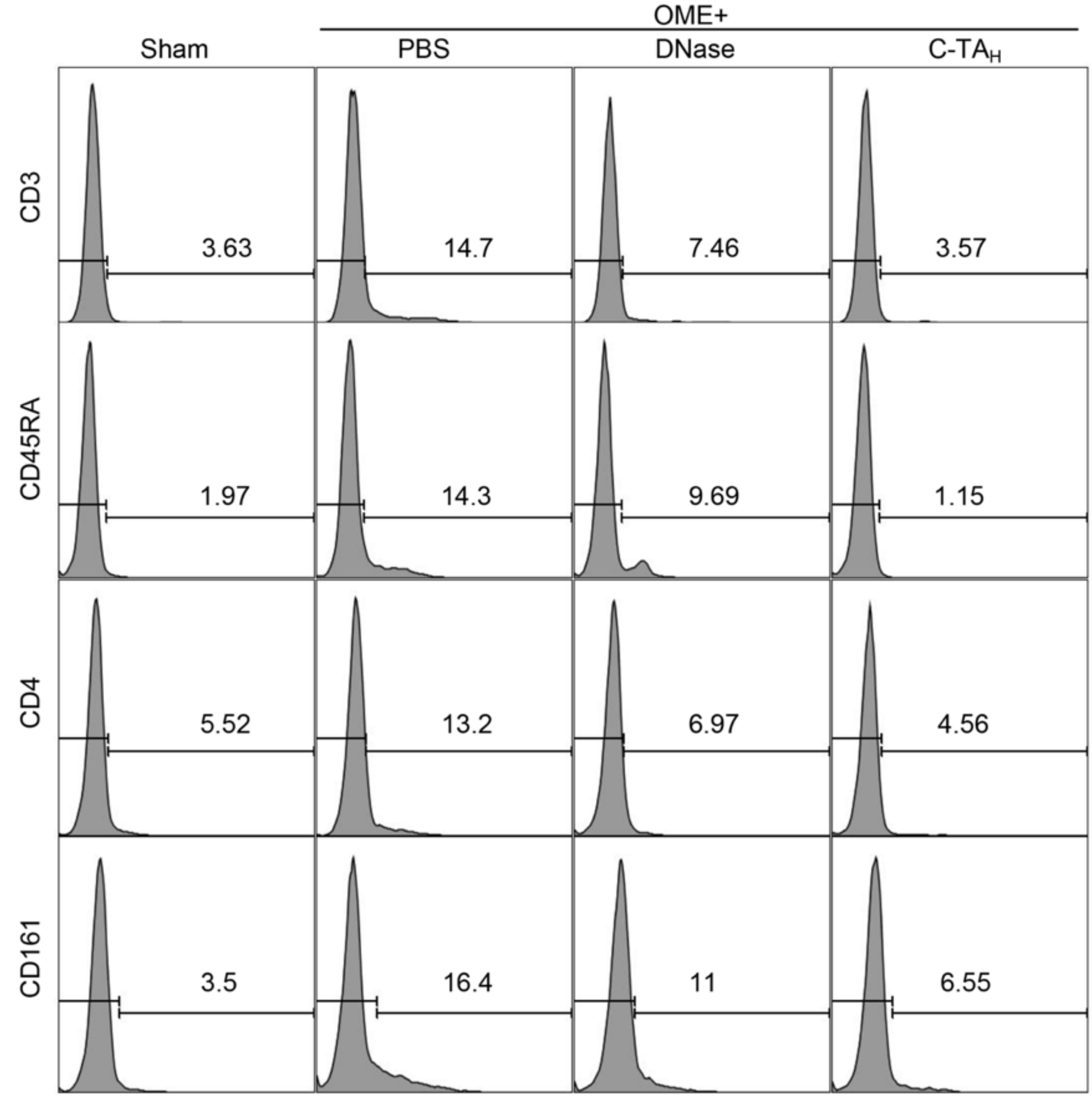

Figure S30. Flow cytometric analysis of immune cell populations in middle ear lavage fluid from rats in different treatment groups. CD3 marks T lymphocytes, CD4 marks helper T cells, CD161 marks natural killer T cells, and CD45RA marks naïve T cells.

18. Veen maps

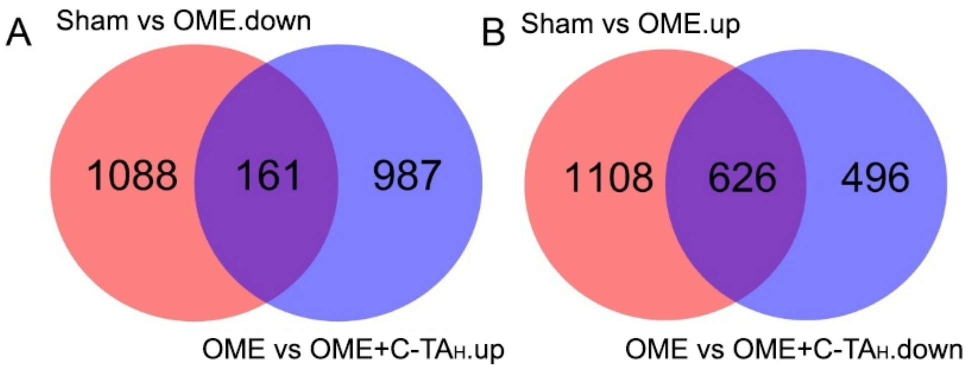

Figure S31. (A) The Venn map of the up-regulated genes (Sham vs OME group), down-regulated genes (OME vs OME+C-TA$_H$ group) and the overlapped genes. (B) the up-regulated genes (Sham vs OME group), down-regulated genes (OME vs OME+C-TA$_H$ group), and the overlapped genes (B).

19. KEGG analysis

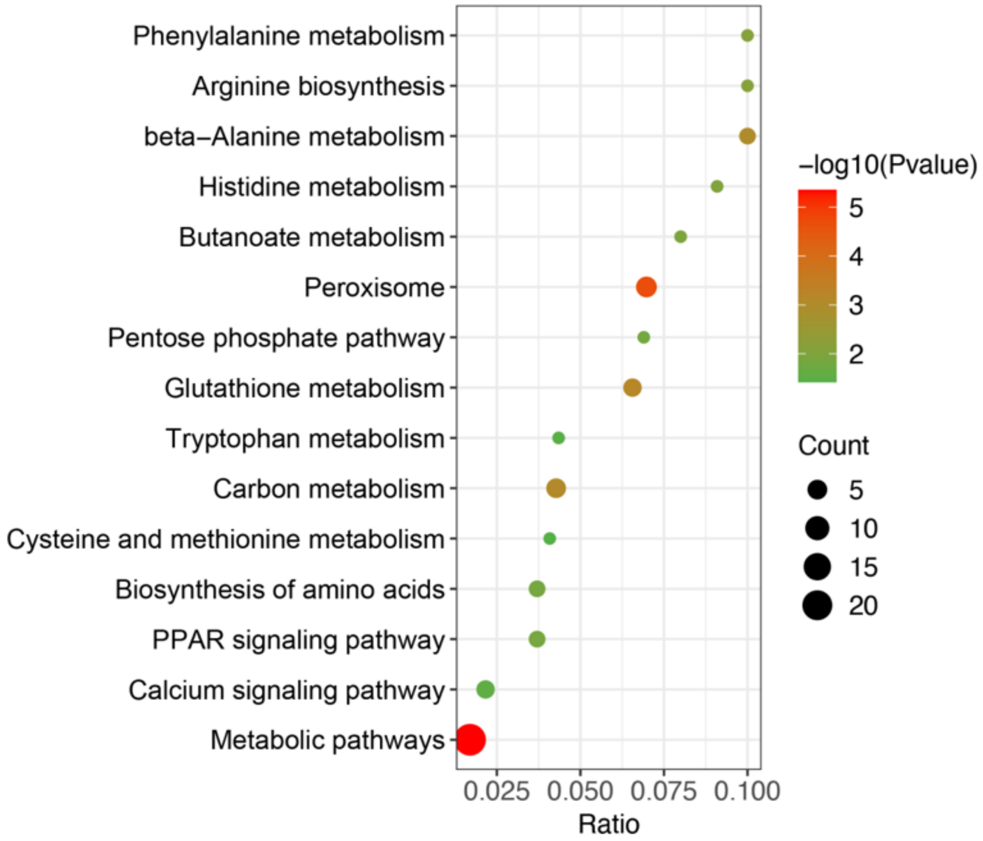

Figure S32. Kyoto Encyclopedia of Genes and Genomes (KEGG) analysis of the 161 genes downregulated in the OME group compared to the Sham group and subsequently upregulated in the OME+C-TA$_H$ group.

20. GO analysis

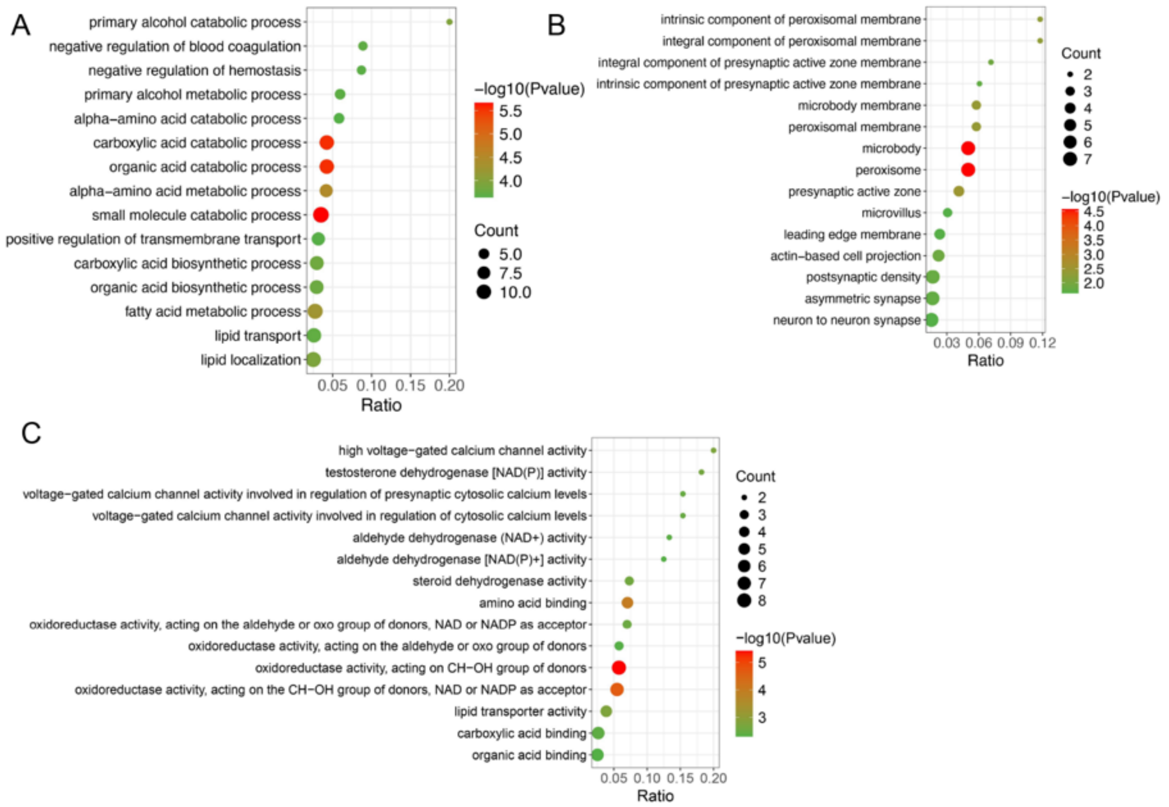

Figure S33. Gene Ontology (GO) term enrichment analyses of 161 genes that were downregulated in the OME group compared to the Sham group and subsequently upregulated in the OME+C-TA$_H$ group. (A) biological process, BP; (B) cellular component, CC; (C) molecular function, MF.

21. GSEA analysis based on KEGG results

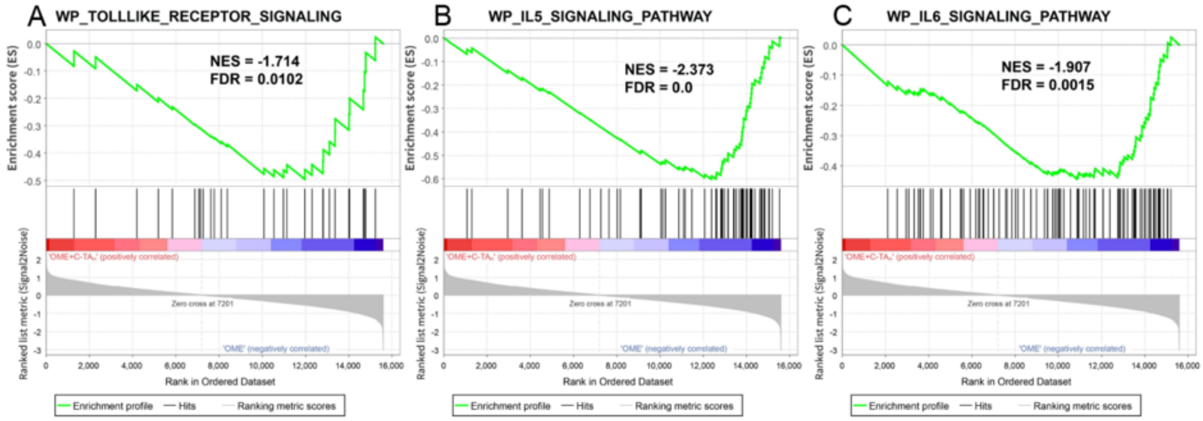

Figure S34. Gene Set Enrichment Analysis (GSEA) based on KEGG results of the 626 overlapped genes that were upregulated in the OME group compared to the Sham group and subsequently downregulated in the OME+C-TA$_H$ group relative to the OME group. (A) Toll-like receptor signaling, (B) IL-5 signaling, and (C) IL-6 signaling pathway.

References

[1] G. Zhang, Y. Cong, F. L. Liu, J. Sun, J. Zhang, G. Cao, L. Zhou, W. Yang, Q. Song, F. Wang, K. Liu, J. Qu, J. Wang, M. He, S. Feng, D. Baimanov, W. Xu, R. H. Luo, X. Y. Long, S. Liao, Y. Fan, Y. F. Li, B. Li, X. Shao, G. Wang, L. Fang, H. Wang, X. F. Yu, Y. Z. Chang, Y. Zhao, L. Li, P. Yu, Y. T. Zheng, D. Boraschi, H. Li, C. Chen, L. Wang, Y. Li, *Nat Nanotechnol* **2022**, 17, 993.

[2] F. Liu, S. Sheng, D. Shao, Y. Xiao, Y. Zhong, J. Zhou, C. H. Quek, Y. Wang, Z. Hu, H. Liu, Y. Li, H. Tian, K. W. Leong, X. Chen, *Nano Lett* **2021**, 21, 2461.
